# E2F1 Mediated Apoptosis Induced by the DNA Damage Response Is Blocked by EBV Nuclear Antigen 3C in Lymphoblastoid Cells

**DOI:** 10.1371/journal.ppat.1002573

**Published:** 2012-03-15

**Authors:** Abhik Saha, Jie Lu, Lise Morizur, Santosh K. Upadhyay, Mahadesh Prasad AJ, Erle S. Robertson

**Affiliations:** Department of Microbiology and Tumor Virology Program of the Abramson Comprehensive Cancer Center, Perelman School of Medicine at the University of Pennsylvania, Philadelphia, Pennsylvania, United States of America; University of Texas Health Science Center San Antonio, United States of America

## Abstract

EBV latent antigen EBNA3C is indispensible for *in vitro* B-cell immortalization resulting in continuously proliferating lymphoblastoid cell lines (LCLs). EBNA3C was previously shown to target pRb for ubiquitin-proteasome mediated degradation, which facilitates G1 to S transition controlled by the major transcriptional activator E2F1. E2F1 also plays a pivotal role in regulating DNA damage induced apoptosis through both p53-dependent and -independent pathways. In this study, we demonstrate that in response to DNA damage LCLs knocked down for EBNA3C undergo a drastic induction of apoptosis, as a possible consequence of both p53- and E2F1-mediated activities. Importantly, EBNA3C was previously shown to suppress p53-induced apoptosis. Now, we also show that EBNA3C efficiently blocks E2F1-mediated apoptosis, as well as its anti-proliferative effects in a p53-independent manner, in response to DNA damage. The N- and C-terminal domains of EBNA3C form a stable pRb independent complex with the N-terminal DNA-binding region of E2F1 responsible for inducing apoptosis. Mechanistically, we show that EBNA3C represses E2F1 transcriptional activity via blocking its DNA-binding activity at the responsive promoters of p73 and Apaf-1 apoptosis induced genes, and also facilitates E2F1 degradation in an ubiquitin-proteasome dependent fashion. Moreover, in response to DNA damage, E2F1 knockdown LCLs exhibited a significant reduction in apoptosis with higher cell-viability. In the presence of normal mitogenic stimuli the growth rate of LCLs knockdown for E2F1 was markedly impaired; indicating that E2F1 plays a dual role in EBV positive cells and that active engagement of the EBNA3C-E2F1 complex is crucial for inhibition of DNA damage induced E2F1-mediated apoptosis. This study offers novel insights into our current understanding of EBV biology and enhances the potential for development of effective therapies against EBV associated B-cell lymphomas.

## Author Summary

Aberrant cellular proliferation due to deregulation of E2F1 transcriptional activity as a result of either genetic or functional alterations of its upstream components is a hallmark of human cancer. Interestingly, E2F1 can also promote cellular apoptosis regardless of p53 status by activating a number of pro-apoptotic genes in response to DNA damage stimuli. Epstein-Barr virus (EBV) encoded essential latent antigen EBNA3C can suppress p53-mediated apoptotic activities. This study now demonstrates that EBNA3C can further impede E2F1 mediated apoptosis by inhibiting its transcriptional ability, as well as by facilitating its degradation in an ubiquitin-proteasome dependent manner. This is the first evidence, which shows through targeting EBNA3C function linked to the E2F1-mediated apoptotic pathway, an additional therapeutic platform could be implemented against EBV-associated human B-cell lymphomas.

## Introduction

The role of the pRb-E2F pathway in the regulation of cell-cycle progression, particularly the G1-S transition, is well established [1]. Several lines of evidence have suggested different roles for individual members of the E2F family of proteins in regulating cell proliferation [2], [3]. There are eight different E2F genes (E2F1-8) belonging to this family in mammals and can be sub-grouped into two classes on the basis of their transcriptional activity [3], [4]. E2F1-3, referred to as the ‘activator E2Fs’, bind to pRb and their ectopic expression was shown to be sufficient for driving cells into S-phase [4]. E2F4-8 largely function as transcriptional repressors and are referred to as the ‘repressor E2Fs’ [4]. The repressor E2Fs can be further divided into two subfamilies. E2F4-5 repress gene expression in an Rb family-dependent manner, whereas E2F6-8 exert transcriptional repression through Rb-independent mechanisms [4].

Interestingly, only E2F1 was shown to play a dual role in controlling both cell growth and apoptosis [2], [5], [6]. For example, elevated expression of E2F1 promotes cell-cycle progression by driving quiescent cells into S phase [7], and in cooperation with activated ras, E2F1 can transform rat embryo fibroblast cells [8]. However, E2F1 expression can also induce apoptosis in the absence of proliferative signals [9]. A physiological role for E2F1-mediated apoptosis has been documented in several studies. E2F1^−/−^ knockout mice develop tumors with high incident rate, signifying that E2F1 is also engaged with growth inhibitory and tumor suppressive activities [10], [11]. Moreover, over-expression of E2F1 in mouse embryonic fibroblasts results in cells entering premature S phase and significant apoptosis [6].

E2F1 mediated apoptosis is known to be associated with both p53 dependent and independent mechanisms [4]. E2F1 accelerates p53 mediated apoptotic activity either by inducing the expression of p19/p14^ARF^, an inhibitor of the Mdm2 ubiquitin ligase that specifically targets p53 for ubiquitin-proteasome mediated degradation or by enhancing p53 phosphorylation [2], [4]. Moreover, E2F1 can also induce apoptosis by transactivating the p53 homologue p73 and Apaf-1 (apoptosis activating factor-1) in response to DNA damage signals [2], [4], [12], [13], [14].

The signaling events that lead to E2F1 induction upon DNA damage response have also been documented [9], [15], [16], [17]. In response to DNA damage, unlike other members of the E2F family, E2F1 is uniquely induced by both ATM (ataxia telangiectasia mutated) and ATR (ATM and Rad3-related) through specific phosphorylation at serine 31 [17]. E2F1 is also shown to be phosphorylated by Chk2 [16]. In general, these phosphorylation events lead to stabilization and activation of E2F1 [16]. In addition to phosphorylation, both acetylation as well as ubiquitination have also been recognized to play an important role in activation and stabilization of E2F1 in response to DNA damage [9], [15], [18]. Thus, it appears that several DNA damage signaling pathways are actively engaged with the induction of E2F1 mediated apoptosis.

EBV is a lymphotropic γ-herpesvirus that asymptomatically persists in more than 90% of the world population [19], [20]. However, EBV intermittently causes a self-limiting disease, infectious mononucleosis in adolescents and has been shown to be associated with the development of several B-cell lymphomas and epithelial cancers primarily in immuno-compromized individuals [19], [21]. *In vitro*, EBV can efficiently transform quiescent B-cells into continuously proliferating lymphoblastoid cell lines (LCLs), providing a surrogate model for EBV associated B-cell tumorigenesis [19], [21], [22]. These latently infected LCLs carry the viral genome as extra-chromosomal episomes that express only a small subset of genes including six nuclear antigens (EBNA- 1, 2, 3A, 3B, 3C and LP), three membrane associated proteins (LMP- 1, 2A, and 2B) and several non-coding RNAs [19], [21]. Genetic studies using recombinant viruses from a number of different groups have established that EBNA1, EBNA2, EBNA3A, EBNA3C and LMP1 are important for EBV mediated transformation of naïve B-cells *in vitro* [19], [21], [23], [24], [25]. Interestingly, EBNA-LP is not absolutely required for *in vitro* B-cell transformation, but necessary for efficient long-term growth of transformed B-cell lines [26].

EBNA3C was initially identified as a transcriptional modulator that can efficiently regulate the transcription of both viral and cellular genes [27], [28], [29]. Coupled with RBP-Jκ, EBNA3C mediated regulation of Notch-induced transcription was shown to be one of the major signaling pathways important for LCL propagation [30], [31], [32]. In addition, EBNA3C was also shown to interact with a wide range of transcription factors and modifiers, such as c-Myc [33], SUMO1 [34], SUMO3 [34], HDAC1 [35], CtBP [36], DP103 [37], p300 [38], prothymosin-α [38], Nm23-H1 [39], p53 [40] and its regulatory proteins Mdm2 [41], ING4 [42] and ING5 [42]. Recently, we showed that EBNA3C can repress p53 dependent apoptotic activity by either blocking its transcriptional activity or recruiting Mdm2 activity for ubiquitin-proteasome mediated degradation [40], [41]. Moreover, EBNA3C attenuates the p53 function through blocking the interaction between p53 and its regulatory proteins, inhibitor of growth family proteins ING4 and ING5 [42].

In this study we address the influence of EBNA3C on suppressing E2F1 mediated apoptosis, independent of p53 in EBV transformed LCLs. We find that EBNA3C can prevent cells from entering E2F1-dependent apoptosis both at early and latent stage of infection and possibly that this effect is critically dependent on the specific interaction between the DNA binding domain of E2F1 and EBNA3C. Regulation of E2F1 mediated apoptosis correlates with EBNA3C-dependent inhibition on E2F1 transcriptional activity at apoptosis related genes, including p73 and Apaf-1. Moreover, EBNA3C specifically targets E2F1 for an ubiquitin-proteasome mediated degradation. Most importantly, we show that E2F1 plays a dual role in regulating LCLs outgrowth. In the presence of growth factors, E2F1 promotes cell proliferation while DNA damage signals can trigger E2F1 mediated apoptosis and cell death. Our data define a new interplay between EBNA3C and E2F1 mediated apoptosis that occurs independently of p53. Overall, this study supports a model where EBNA3C can antagonize the apoptotic properties of both E2F1 and p53 to maintain EBV transformed cells in a continuous state of growth stimulation.

## Materials and Methods

### Cell cultures, plasmids, antibodies and transfection

HEK 293, HEK 293T, and both p53 and pRb null Saos-2 cells were maintained as described previously [41], [43]. Burkitt's lymphoma cell lines DG75, Ramos, BJAB, BJAB stably expressing EBNA3C clones (E3C7 and E3C10) and the *in vitro* EBV-transformed lymphoblastoid cell lines (LCL1 and LCL2) have been previously described [40], [41], [43].

pEGFP-EBNA3C expressing GFP-fused wild-type EBNA3C (residues 1–992) and myc-tagged EBNA3C constructs (expressing amino acids 1–992, 1–365, 366–620, 621–992, 1–159, 1–129 and 1–100) in pA3M vector have been previously mentioned [41], [43]. Other myc-tagged truncated EBNA3C constructs (encoding amino acids 1–300, 1–250, 50–300, 130–300, 160–300, 200–300, 621–950, 621–850, 621–800, 621–750 and 700–900) were generated by PCR amplification followed by directional cloning in pA3M vector at EcoRI and NotI restriction sites. pGEX-E2F1 plasmid expressing GST-fused wild-type E2F1 (encoding residues 1–437) was kindly provided by Pradip Raychaudhuri (University of Illinois, Chicago, IL, USA) and used to generate wild-type and different truncated versions of E2F1 (expressing residues 1–437, 1–400, 1–310, 1–243, 243–437 and 1–150) fused with either C-terminal flag-epitope or N-terminal GST-tag into pA3F [33], and modified pGEX-2TK vectors [43], respectively at EcoRI and NotI restriction sites. E2F1 reporter plasmids (pGL2-basic-3X-WT-E2F1-Luc and pGL2-basic-3X-Mut-E2F1-luc) containing either three wild-type E2F1 binding sites (CTGCAATTTCGCGCCAAACTT) or three mutant E2F1 binding sites (CTGCAATTGCTCGACCAACTT) fused upstream of the luciferase gene were generously provided by Stefan Gaubatz (Philipps University, Marburg, Germany) [44]. Human wild-type p73 [45] and Apaf-1 [46] promoters linked to luciferase gene were obtained as kind gifts from Mirko Marabese (Istituto di Ricerche Farmacologiche “Mario Negri”, Milan, Italy) and Kristian Helin (European Institute of Oncology, Milan, Italy), respectively. Lentiviral packaging vectors, sh-RNA expressing lentiviral vectors directed against either EBNA3C (pGIPZ-Sh-E3C.1) or control that lacks any complementary sequence in the human genome (pGIPZ-Sh-Con) were previously described [43]. Sh-RNA directed against E2F1 was cloned into pGIPZ vector (Open Biosystems, Inc. Huntsville, AL). The sense strand of E2F1 sh-RNA sequence #1 is 5′-tcgagtgctgttgacagtgagcgaGACTGTGACTTTGGGGACCTtagtgaagccacagatgtaAGGTCCCCAAAGTCACAGTCgtgcctactgcctcggaa-3′ [47]. The sense strand of another E2F1 sh-RNA sequence #2 is 5′-tcgagtgctgttgacagtgagcgaGACTGTGACTTTGGGGACCTtagtgaagccacagatgtaAGGTCCCCAAAGTCACAGTCgtgcctactgcctcggaa-3′ [47]. Upper-case letters indicate 20-nucleotide (nt) E2F1 target sequence and lowercase letters indicate hairpin and sequences necessary for the directional cloning into pGIPZ at Xho I and Mlu I restriction sites. All constructs and mutations were verified by DNA sequencing (University of Pennsylvania DNA sequencing facility).

Rabbit polyclonal antibodies reactive to E2F1 (C-20), Apaf-1 (H-324), Cyclin E (M-20) Ubiquitin (FL-76); goat polyclonal antibody against p73 (S-20); and mouse monoclonal antibodies against PARP1 (F-2) and GFP (F56-BA1) were obtained from Santa Cruz Biotechnology, Inc. (Santa Cruz, CA). Mouse monoclonal antibody against GAPDH was bought from US-Biological Corp. (Swampscott, MA). Mouse monoclonal antibodies reactive to myc epitope (9E10), flag epitope (M2), EBNA3C (A10), LMP1 (S12) and EBNA2 (PE 2) have been described previously [40], [41], [43], [48], [49]. Rabbit polyclonal antibody specific for EBNA3C was obtained from Cocalico Biologicals, Inc. (Reamstown, PA) and has been described previously [35].

Adherent cells were transiently transfected either by electroporation with a Bio-Rad Gene Pulser II electroporator as previously described [40], [41], [43], or using Lipofectamine 2000 (Invitrogen, Carlsbad, CA) according to manufacturer's protocol. LCLs were transfected with 50 ìg of plasmids via electroporation (Bio-Rad Gene Pulser II; 230 V, 975 µF). Transfected LCLs were cultured in RPMI medium with 10% FBS for 48 h.

### Immunoprecipitation (IP), Western blotting (WB), and Immuno-fluorescence (IF)

10 million transiently transfected cells or 50 million B-cells were harvested, washed with ice cold PBS and subsequently lysed in 0.5 ml ice cold RIPA buffer [1% Nonidet P-40 (NP-40), 10 mM Tris pH 8.0, 2 mM EDTA, 150 mM NaCl, supplemented with protease inhibitors (1 mM phenylmethylsulphonyl fluoride (PMSF), 1 µg/ml each aprotinin, pepstatin and leupeptin]. Lysates were precleared with normal control serum plus 30 µL of a 1∶1 mixture of Protein-A/G Sepharose beads (GE Healthcare Biosciences, Pittsburgh, PA) for 1 h at 4°C. Unless and otherwise stated, approximately 5% of the precleared lysate was saved for input control and the protein of interest was captured by rotating the remaining lysate with 1 µg of specific antibody overnight at 4°C. Immuno-complexes were captured with 30 µl Protein-A/G beads, pelleted and washed 5X with ice cold RIPA buffer.

Input lysates and IP complexes were boiled in laemmli buffer [50], fractionated by SDS-PAGE and transferred to a 0.45 µm nitrocellulose membrane for WB analyses. The membranes were then probed with specific antibodies followed by incubation with appropriate infrared-tagged secondary antibodies and viewed on an Odyssey imager. Image analysis and quantification measurements were performed using the Odyssey Infrared Imaging System application software (LiCor Inc., Lincoln, NE).

IF experiments were performed essentially as described previously [41], [43]. Briefly, Saos-2 cells plated on coverslips were transfected with expression vectors as indicated, using Lipofectamine 2000. After 36 h of transfection, cells were fixed by ice cold acetone: methanol mixture (1∶1) for 10 min at −20°C. LCLs were air-dried and fixed similarly. Ectopically expressed E2F1 was detected using M2-antibody, and GFP-EBNA3C was detected by GFP fluorescence. In LCLs, endogenously expressed EBNA3C and E2F1 proteins were detected using their specific antibodies. The slides were examined with a Fluoview FV300 confocal microscope (Olympus Inc., Melville, NY).

### Purification of GST-proteins and GST-pulldown assays

*Escherichia coli* BL21 competent cells were transformed with plasmids for each Glutathione S-transferase (GST) fusion protein and protein complexes containing the tagged proteins were purified essentially as previously described [40], [41], [43].

For *in vitro* GST-pulldown experiments, GST fusion proteins were incubated with *in vitro*-translated ^35^S-labeled protein in binding buffer (1× phosphate-buffered saline [PBS], 0.1% NP-40, 0.5 mM dithiothreitol [DTT], 10% glycerol, supplemented with protease inhibitors). *In vitro* translation was done with the TNT T7 Quick Coupled Transcription/Translation System (Promega Inc., Madison, WI) according to the manufacturer's instruction.

### Promoter assays

Promoter assays were performed as previously described with few modifications [40], [42]. Briefly, either 10 million Saos-2 (pRb^−/−^) cells were transiently transfected by electroporation with indicated plasmids. Cells were additionally transfected with pEGFP-C1 and pCMV-βgal constructs for measuring the transfection efficiency. After 36 h of transfection, cells were harvested, lysed in reporter lysis buffer (Promega Inc., Madison, WI) and the luciferase as well as β-galactosidase activities were measured using either an LMaxII384 luminometer (Molecular Devices, Sunnyvale, CA) or VERSAmax microplate reader (Molecular Devices, Sunnyvale, CA), respectively. The results are shown as representation of duplicate experiments.

### ChIP assay

Chromatin immunoprecipitation (ChIP) assay was performed as previously described [51]. Briefly, 20 million HEK293 cells were transiently transfected by electroporation with E2F1 reporter plasmid and expression vectors for flag-E2F1 and myc-EBNA3C. 36 h of post-transfection, cells were cross-linked by 1% formaldehyde, harvested, sheared DNA to an average length of 700 bp by sonication. Cross-linked DNA was immunoprecipitated by anti-flag antibody and subjected for PCR analysis using primers designed either for E2F1-responsive promoter fused with luciferase gene or control SV-40 promoter region of pGL2-basic vector. Primers for E2F1-promoter: 5′-TTGCCGATTTCGGCCTATTG-3′ and 5′-CATCCTCTAGAGGATAGAATGG-3′; primers for SV-40 promoter: 5′-CGTTGTTGTTTTGGAGCACGGA-3′ and 5′-TTGGACTTTCCGCCCTTCTTG-3′. For EBV-positive cells (LCL1 with either Sh-Con or Sh-E3C), 50 million cells were collected, immunoprecipitated with either control rabbit antibody or E2F1 specific antibody and processed as above. Eluted DNA fragments were analyzed by subsequent PCR with primers specific for the p73 and Apaf-1 human promoters. Primers used in this assay are: for p73 promoter: 5′- TGAGCCATGAAGATGTGCGAG-3′ and 5′- GCTGCTTATGGTCTGATGCTTATG-3′ [52]; for Apaf-1 promoter: 5′-GCCCCGACTTCTTCCGGCTCTTCA-3′ and 5′-GGAGCTGGCAGCTGAAAGACTC-3′ [53].

### Colony formation assay

10 million Saos-2 (p53^−/−^) cells were transfected by electroporation with indicated expression plasmids for flag-E2F1 and myc-EBNA3C. Cells were additionally transfected with a GFP expression vector (pEGFP-C1, BD Biosciences Clontech). After 24 h of transfection the cells were serum starved (DMEM with 0.1% FBS) with 5 µM etoposide (MP Biomedicals) for 12 h, followed by selection with DMEM supplemented with 5 mg/ml G418 (Invitrogen, Inc., Carlsbad, CA) for 2 weeks. After selection, cells were fixed on the plates with 4% formaldehyde and scanned for GFP expressed colonies using Typhoon 9410 imaging system (GE Healthcare Biosciences, Pittsburgh, PA). The area of the colonies (pixels) in each dish was calculated by Image J software (Adobe, San Jose, CA). The data are shown as the average and standard deviation of two independent experiments.

### Detection of apoptosis

Saos-2 cells were transfected and selected as above. Approximately 2 million puromycin selected LCLs, EBV positive LCLs (LCL1 and LCL2) or EBV negative Burkitt's lymphoma cell lines DG75 and Ramos were exposed to serum starvation (RPMI with 0.1% FBS) and 5 µM etoposide treatment for 12 h. For E2F1 knockdown LCLs, cells were additionally treated with an increasing concentration of etoposide (0, 5, 10 and 20 µM) for 12 h in absence of serum. Subsequently cells were collected, fixed in cold 70% ethanol for 2 h at −20°C, washed with 1× PBS and stained with PI staining buffer (10 mM Tris, pH 7.5; 0.2 mg/ml RNase A, and 50 mg/ml propidium iodide) for 2 h in the dark at room temperature. The stained cells were analyzed using FACScan (BD Biosciences, San Jose, CA) and FlowJo (Tree Star, Inc. Ashland, OR) software.

### Proliferation assay

Approximately 1×10^5^ Saos-2 cells selected for either flag-E2F1 or flag-E2F1 plus myc-tagged EBNA3C constructs (either wild-type, residues 1–992 or truncated version, residues 366–620) were plated into each well of the 6-well plates, exposed to serum starvation (DMEM with 0.1% FBS) and 5 µM etoposide treatment for 12 h, followed by culturing in regular medium for 6 days at 37°C. Viable cells from each well were counted by trypan blue exclusion method daily using a Bio-Rad TC10 Automated cell counter. In parallel assays cells were harvested, lysed in RIPA buffer and subjected for western blot analyses using indicated antibodies.

For B-cells, approximately 1×10^5^ cells (Ramos, DG75, LCL1 and LCL2) were plated into each well of the 6-well plates and cultured at 37°C in either complete RPMI medium or RPMI supplemented with 0.1% FBS plus 5 µM etoposide. Cells were counted similarly for 6 days. Both experiments were performed in duplicate and were repeated two times.

### TUNEL assay

The TUNEL assay was performed by using an In Situ Cell Death Fluorescein Detection kit (Roche, Indianapolis, IN) in accordance with the manufacturer's instructions. Saos-2 cells transfected with the indicated expression plasmids for flag-E2F1 and myc-EBNA3C were selected for 2 weeks with G418. After 12 h treatment with 0.1% FBS containing DMEM plus 5 µM etoposide for 12 h, terminal transferase reaction was performed on 1∶1 methanol∶acetone fixed cells in 6 well plates. Apoptosis was measured by counting green cells using a Fluoview FV300 confocal microscope (Olympus Inc., Melville, NY).

### Lentivirus production and transduction of LCLs

Lentivirus production and transduction of EBV-transformed B-cells (LCLs) were essentially carried out as previously described [43].

### Infection of PBMCs with wild-type or ΔEBNA3C BAC GFP-EBV

Generation of BAC GFP-EBV was previously described [22]. EBNA3C mutant (BAC GFP-EBVΔE3C) was generated from wild-type BAC GFP-EBV construct. For the generation of BAC GFP-EBVΔ E3C we selected the region from 91822 to 102891 bp from wild-type BAC GFP-EBV plasmid and deleted the corresponding EBNA3C region from 98370–101424 bp. Southern blot analysis and junction PCR was performed to confirm the mutant generation.

For infection, peripheral blood mononuclear cells (PBMC) from healthy donors were obtained from University of Pennsylvania Immunology Core. As previously described [22], [43], approximately 10 million PBMC were mixed with virus (either wild-type or mutant) supernatant in 1 ml of RPMI 1640 with 10% FBS for 4 hr at 37°C in 6-well plates. Cells were centrifuged for 5 min at 500 g, discarded the supernatant, pelleted cells and resuspended in 2 ml of complete RPMI 1640 medium in 6 well plates. EBV GFP expression visualized by fluorescence microscopy was used to quantify infection. The protein and mRNA level of the infected cells was detected after indicated days of post-infection.

### Real time quantitative PCR

Total RNA was isolated by using TRIzol reagent according to the instructions of the manufacturer (Invitrogen, Inc., Carlsbad, CA). cDNA was made by using a Superscript II reverse transcriptase kit (Invitrogen, Inc., Carlsbad, CA) according to the instructions of the manufacturer. The primers were for *E2F1*, 5′- GGCCAGGTACTGATGGTCA-3′, and 5′-GACCCTGACCTGCTGCTCT-3′, for *p73* 5′-CCCCATCAGGGGAGGTG-3′, and 5′-AGGGGACGCAGCGAAAC-3′, for *Apaf-1* 5′- CCTCTCATTTGCTGATGTCG-3′ and 5′-TCACTGCAGATTTTCACCAGA-3′, for *cyclin E* 5′-GTTATAAGGGAGACGGGGAG-3′ and 5′-TGCTCTGCTTCTTACCGCTC-3′, and for *GAPDH* 5′-TGCACCACCAACTGCTTAG-3′ and 5′-GATGCAGGGATGATGTTC-3′. Quantitative real-time PCR analysis was done using StepOnePlus Real-Time PCR System (Applied Biosystems, Foster City, CA) in triplicate as previously described [43].

### Oligo-pulldown assay

100 µg of cell extracts from Saos-2 cells transfected with flag-E2F1 with or without myc-EBNA3C expression vector were incubated with 200 ng of the indicated biotinylated oligonucleotides (wild-type or mutant) immobilized with streptavidin accordingly to the manufacturer protocol, in the absence or presence of a 200 molar excess of the corresponding non biotinylated oligonucleotide. Oligonucleotide-bound E2F1 protein was washed 3X with RIPA buffer and detected by western blotting using anti-flag antibody. The band intensities were scanned using KODAK 1D Image Analysis software. The oligonucleotides [9] used in this assay are: for p73 wild-type promoter 5′-GCCGCCTTTTGGCGCGCGTCGCTCCTGCAGAG-3′; for p73 mutant promoter 5′-GCCGCCTTGTAGAGTGCGTCGCTCCTGCAGAG-3′; for Apaf-1 wild-type promoter 5′-AGTCAAATCCCGCCGGATCCACCCAGCCCGGA-3′; for Apaf-1 mutant promoter 5′-AGTCAAATTCAGTCAGATCCACCCAGCCCGGA-3′.

### Stability assay

10 million Saos-2 (pRb^−/−^) cells were transiently transfected using electroporation with flag-tagged E2F1 with or without myc-tagged EBNA3C expression plasmids. Cells were additionally trasfected with GFP-expressing plasmid for measuring the trasfection efficiency. After 36 hours transfection, cells were treated with 40 µg/ml cyclohexamide (CalBiochem, Gibbstown, NJ). For LCLs, 20 million cells were treated with 100 µg/ml cyclohexamide (CalBiochem, Gibbstown, NJ) either in normal serum medium or in 0.1% FBS containing DMEM plus 5 µM etoposide. Subequently, lysates were prepared at indicated time periods and subjected to immunoblot analyses with appropriate antibodies. Band intensities were quantitated using Odyssey 3.0 software provided by Odyssey imager (LiCor Inc., Lincoln, NE).

### *In vivo* ubiquitination assay

15 million HEK 293 cells were transfected by electroporation with appropriate plasmids expressing HA-Ub, flag-E2F1 and myc-EBNA3C. Cells were incubated for 36 h and pretreated for an additional 12 h with 20 µM MG132 (Enzo Life Sciences, Inc. Farmingdale, NY) before harvesting. Flag-E2F1 was immunoprecipitated with M2 antibody and resolved by SDS-PAGE. The extent of ubiquitination of flag-tagged proteins was determined by western blot analysis using the anti HA-antibody (12CA5). For LCLs (Sh-Control and Sh-EBNA3C), cells were treated with 40 µM MG132 (Enzo Life Sciences, Inc. Farmingdale, NY) and immunoprecipitated with anti-E2F1 polyclonal antibody and subjected for western blot with indicated antibodies.

## Results

### EBNA3C interacts with E2F1 in a pRb independent manner

Several lines of evidence suggest that EBNA3C manipulates G1 cell-cycle restriction point through disruption of Cyclin/CDK-pRb-E2F pathway in EBV infected human cells [43], [54], [55], [56], [57]. For example, EBNA3C directly targets pRb, but not other pocket family proteins including p107 and p130, for ubiquitin-proteasome mediated degradation [54], relieving the negative regulatory pressure on E2F transcriptional factors to facilitate the G1 to S transition [43], [57]. It is therefore tempting to investigate whether or not EBNA3C has any influence in modulating functions of E2F1, the major transcriptional factor in E2F family and whose active participation in both cell-proliferation and apoptosis regulation is unquestionable.

**Figure 1:**
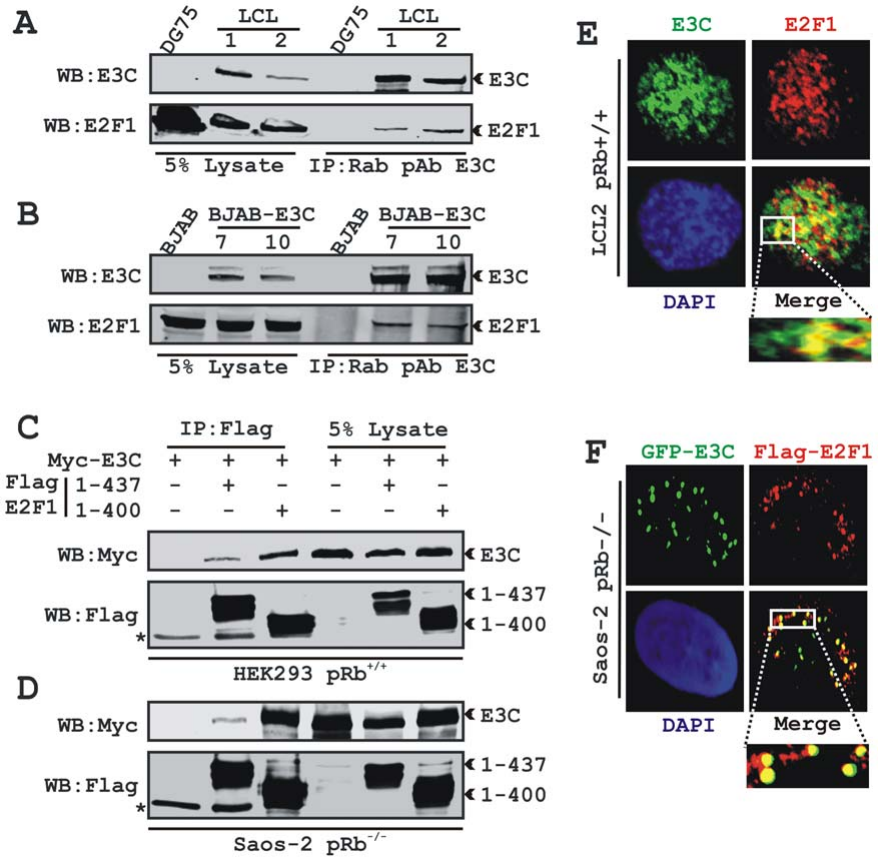
EBNA3C forms a pRb independent complex with E2F1. 50 million A) DG75 and two LCL clones (LCL1 and 2) and B) BJAB and two BJAB stable clones expressing EBNA3C (E3C7 and E3C10) were subjected to immunoprecipitation (IP) with EBNA3C specific rabbit antibody. Samples were resolved by SDS-PAGE and detected by western blot (WB) for the indicated proteins by stripping and reprobing the same membrane. 10 million C) HEK293 (pRb^+/+^) or D) Saos-2 (pRb^−/−^) cells were co-transfected with plasmids expressing myc-EBNA3C either in the presence of vector control, wild-type flag-E2F1 (residues 1–437) or pRb binding deficient flag-E2F1 (residues 1–400) as indicated. At 36 h post-transfection, cells were harvested, lysed in RIPA buffer and IP with flag-antibody. Samples were western blotted (WB) with the indicated antibodies. The asterisks indicate the immunoglobulin bands. E) EBV transformed cells LCL2 were plated and air-dried onto slides. F) Saos-2 (pRb^−/−^) cells plated on coverslips were co-transfected with plasmids expressing GFP-EBNA3C with flag-E2F1 using Lipofectamine 2000 as per manufactures instructions. E) Endogenously and F) ectopically expressed E2F1 were detected by either specific rabbit antibody (C-20) or mouse M2-antibody, respectively, followed by specific anti-Alexa Fluor 594 2^0^ antibody (red). A) Endogenous EBNA3C in EBV positive LCLs was detected using an EBNA3C-specific antibody (A10 ascites) followed by 2^0^ antibody anti-mouse Alexa Fluor 488 (green). Ectopically expressed GFP-EBNA3C in Saos-2 cells was detected by GFP fluorescence. The nuclei were subsequently stained with DAPI and the images were captured using an Olympus confocal microscope. All panels are representative pictures from approximately 100 cells of 10 different fields of three independent experiments.

First, we determined whether EBNA3C can form a complex with E2F1 in EBV infected human B-cells. Endogenously expressed EBNA3C was immunoprecipitated from two EBV-transformed lymphoblastoid cell lines (LCL1 and LCL2) or a Burkitt lymphoma (BL) cell line -BJAB stably expressing EBNA3C (E3C7 and E3C10) using an EBNA3C reactive rabbit polyclonal antibody, and co-immunoprecipitation (co-IP) of E2F1 was monitored by immunoblotting using an E2F1 specific antibody (Figure 1A and 1B, respectively). EBV-negative BL lines DG75 and BJAB were used as controls (Figure 1A and 1B, respectively). The results clearly demonstrated that EBNA3C formed a stable complex with E2F1 in human cells (Figure 1A and 1B). Virtually identical results were obtained when we used a different EBNA3C reactive mouse monoclonal antibody (A10) for co-immunoprecipitation experiments using these cell lines (Figure S1A and S1B). However, since all these B-cells have endogenous pRb expression, we could not rule out the possibility that pRb could serve as a bridging molecule between EBNA3C and E2F1 binding interface.

In order to validate whether this interaction between EBNA3C and E2F1 is either pRb dependent or independent, we next performed binding experiments using two different strategies. We utilized a mutant E2F1 construct (expressing residues 1–400) lacking the pRb interaction domain at the C-terminal region and a pRb-deficient cell line, Saos-2 (pRb^−/−^). Both HEK 293 (pRb^+/+^) and Saos-2 (pRb^−/−^) cells transiently expressing myc-EBNA3C in the presence of either empty vector, or flag-tagged wild-type E2F1 (residues 1–437) or flag-tagged E2F1 mutant (residues 1–400) constructs were harvested after 36 h of transfection and subsequently subjected for IP with anti-flag antibody (Figure 1C and 1D). HEK 293 (pRb^+/+^) cells stably express adenovirus E1A; however, the endogenous pRb-E2F1 complex was shown to be resistant to E1A-mediated disruption [58]. The results showed that EBNA3C was clearly co-immunoprecipitated with both wild-type and mutant E2F1 but not with the vector control in both pRb^+/+^ and pRb^−/−^ cell lines (Figure 1C and 1D, respectively). Importantly, the mutant E2F1 (residues 1–400) bound to EBNA3C with stronger association compared to wild-type protein (Figure 1C and 1D, compare lanes 2 and 3). Perhaps, deletion of the pRb binding region at the C-terminal domain of E2F1 leads to a conformational change of overall E2F1 secondary and tertiary structure, which further allows greater access to EBNA3C's binding site(s). Analysis of both endogenous as well as ectopic expression data strongly demonstrated that EBNA3C forms a pRb-independent complex with E2F1.

For additional support of the binding data and to visualize the sub-cellular localization pattern of E2F1 in the presence of EBNA3C, colocalization experiments were performed with an EBV-transformed cell line, LCL2 (pRb^+/+^) (Figure 1E). Immunofluorescence staining using antibodies specific to E2F1 and EBNA3C proteins demonstrated that both proteins had distinctive nuclear staining with a speckled pattern (Figure 1E). The results showed that E2F1 partly colocalized with EBNA3C in human cells, as visualized by yellow fluorescence when both signals were merged (Figure 1E). The colocalization study was further extended using ectopically expressed flag-tagged E2F1 with the GFP-tagged EBNA3C in Saos-2 (pRb^−/−^) cells. The results showed that E2F1 noticeably colocalized with EBNA3C, as indicated by the merged yellow fluorescence signals within the nucleus (Figure 1F). Further, these colocalization data suggested that EBNA3C shares similar nuclear compartments with E2F1 in a pRb independent manner.

### E2F1 binds to two distinct regions located at the N- and C-terminal domains of EBNA3C

**Figure 2:**
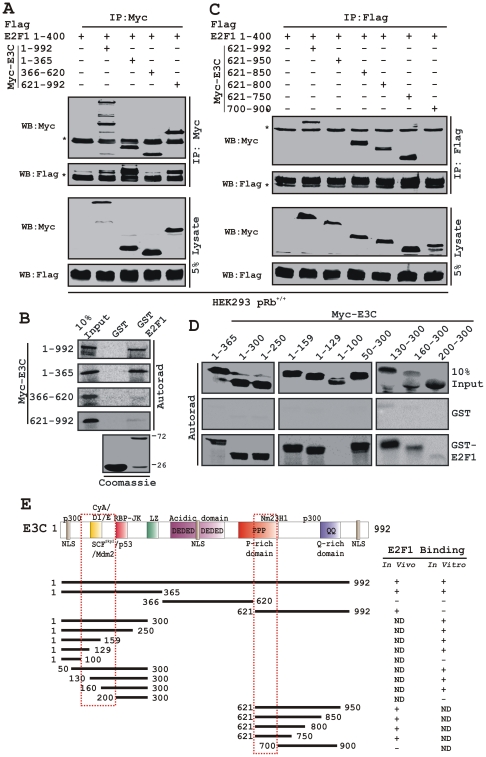
Both N- and C-terminal domains of EBNA3C bind to E2F1. A and C) 10 million HEK293 (pRb^+/+^) cells were co-transfected with plasmids expressing pRb binding deficient flag-E2F1 (residues 1–400) in presence of different myc-EBNA3C constructs as indicated. Cells were harvested, lysed and immunoprecipitated (IP) with anti-flag antibody (M2). Samples were resolved by a 9% SDS-PAGE and detected by western blot (WB) for the indicated proteins by stripping and reprobing the same membrane. B and D) Wild-type or different truncated mutant expression plasmids of EBNA3C were *in vitro* translated in presence of S^35^-Met as per manufacturer's instructions. After preclearing of all S^35^-radiolabeled translated proteins with GST-beads for 1 h at 4°C, samples were subjected to GST-pull-down by incubating with either recombinant GST alone or wild-type GST-E2F1 protein as indicated. Reactions were resolved by a 9% SDS-PAGE, exposed to phosphoimager plate for overnight and scanned using Typhoon 9410 imaging system. Coomassie staining of a parallel SDS-PAGE resolving purified GST-proteins is shown at the bottom panel of B. E) The schematic illustrates different structural and interaction domains of EBNA3C and summarizes the binding studies between different domains of EBNA3C with E2F1. +, binding; −, no binding. ND, not determined. NLS, nuclear localization signal. The asterisks indicate the immunoglobulin bands.

We wanted to determine the functional residues of EBNA3C that specifically interact with E2F1. Two successive binding experiments were performed with different truncated polypeptides of EBNA3C covering the entire length of the molecule (residues 1–992, 1–365, 366–620 and 621–992). First, HEK 293 cells were transfected with flag-tagged E2F1 (residues 1–400) in combination with either the control vector or aforementioned myc-EBNA3C expression constructs. The results showed that E2F1 (residues 1–400) was co-immunoprecipitated with both EBNA3C N-terminal (residues 1–365) and the C-terminal (residues 621–992) domains along with the full-length protein (residues 1–992) (Figure 2A). No co-immunoprecipitation was observed with either the vector control or EBNA3C middle region (residues 366–620) indicating a strong level of specificity of this experiment (Figure 2A).

Next, in order to support the abovementioned binding study, an *in vitro* GST pulldown experiment was conducted using similar EBNA3C fragments. Bacterially purified recombinant GST and GST-E2F1 proteins were incubated with different *in vitro*-translated, S^35−^Met radiolabeled fragments of EBNA3C (residues 1–365, 366–620 and 621–992) using T7 TNT coupled transcription-translation system [41], [59]. Interestingly, in contrast to the immunoprecipitation assay (Figure 2A) the *in vitro* binding assay (Figure 2B) showed a different trend. The results showed that only the N-terminal domain of EBNA3C (residues 1–365) along with the full-length molecule strongly bound with GST-E2F1 (Figure 2B). However, the remaining EBNA3C domains starting from 366 to 992, including the GST control, showed little or no binding (Figure 2B). A parallel Coomassie blue-stained gel showed the amounts of recombinant GST proteins employed in this assay (Figure 2B). The results implicated that the N-terminal residues of EBNA3C is likely to interact directly with E2F1 while the C-terminal region associates with a complex which includes E2F1 in cells (Figure 2A and 2B). However, we could not entirely rule out the possibility that the N-terminal domain of EBNA3C may also form a complex with E2F1 utilizing a protein present in the rabbit reticulocyte lysate as a potential bridging factor.

Earlier studies have shown that both the N- and C-terminal domains of EBNA3C are important to interact with a number of critical cell cycle-regulatory molecules [33], [38], [43], [56], [60], [61]. To narrow down the interacting domain within the N- as well as C-terminal regions of EBNA3C, two successive binding assays were performed using a series of truncated EBNA3C fragments. First, an *in vivo* immunoprecipitation experiment was set up by co-transfecting a flag-tagged E2F1 (residues 1–400) expressing construct with either empty vector or plasmid DNA expressing myc-tagged C-terminal truncated fragments of EBNA3C in HEK 293 cells. The results from immunoprecipitation using anti-flag antibody showed that the C-terminal residues starting from 621 to 700 of EBNA3C is capable of associating with E2F1 (residues 1–400). EBNA3C residues 621–992, 621–950, 621–850, 621–800 and 621–750 were co-immunoprecipitated with flag-E2F1 (residues 1–400), while no co-immunoprecipitation was observed with either the vector control or EBNA3C residues 700–900 (Figure 2C).

Further, to map within the N-terminal domain of EBNA3C (residues 1–365) an *in vitro* GST-pull down experiment was conducted using a series of small truncations of N-terminal EBNA3C (Figure 2D). In vitro precipitation experiments with recombinant GST-E2F1 showed strong interaction with EBNA3C residues 1–365, 1–300, 1–250, 1–159, 1–129, 50–300, 130–300 and 160–300 (Figure 2D) but not with EBNA3C residues 1–100 and 200–300 (Figure 2D). All fragments of EBNA3C failed to interact with the GST control, strongly suggesting that the observed binding was specific for E2F1 (Figure 2D). The results demonstrated that E2F1 interacts with two distinct regions of EBNA3C, one at N-terminal residues 100–200 and another at residues 621–700 (Figure 2E).

### The N-terminal DNA binding domain of E2F1 is responsible for interaction with EBNA3C

**Figure 3:**
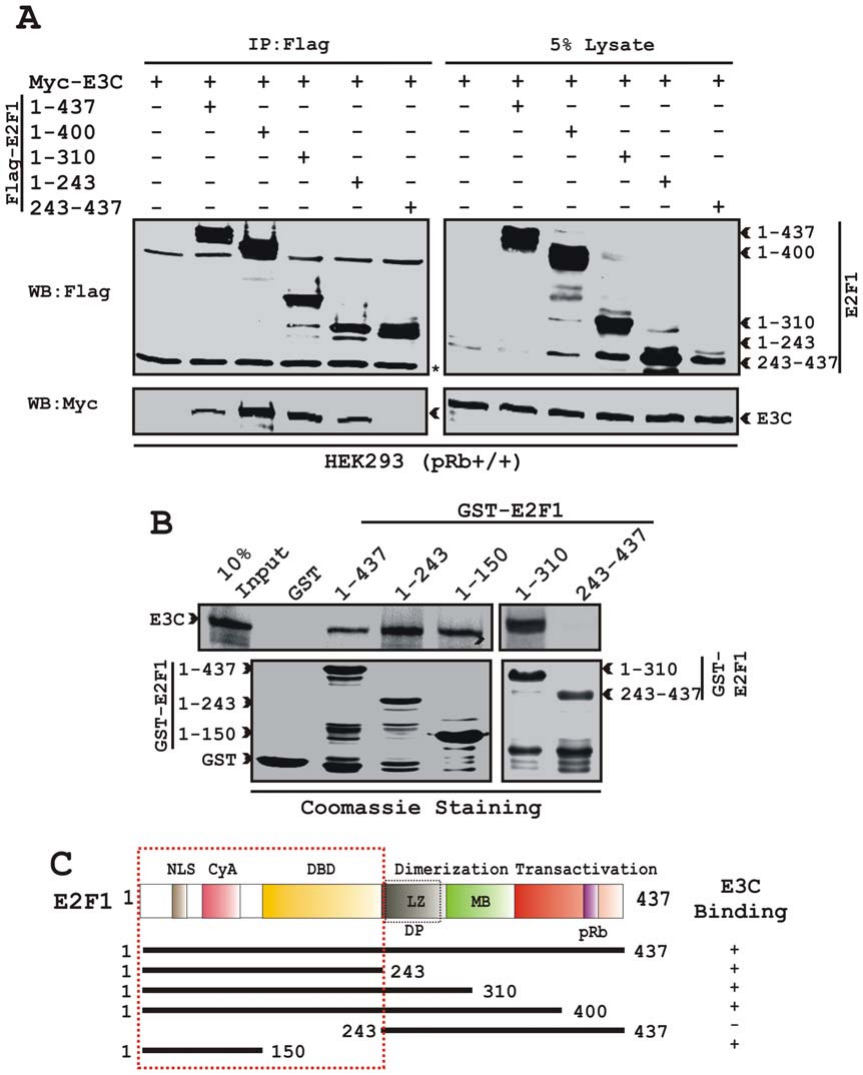
EBNA3C binding region is located at the N-terminal DNA binding domain of E2F1. A) 10 million HEK293 (pRb^+/+^) cells were co-transfected with myc-EBNA3C expressing construct with plasmids expressing either vector control or different truncated versions of E2F1 as indicated. After 36 h of transfection, cells were harvested, lysed and immunoprecipitated (IP) with anti-flag antibody (M2). Samples were resolved by a 9% SDS-PAGE and detected by western blot (WB) for the indicated proteins by stripping and reprobing the same membrane. B–C) GST-fused wild-type or different truncated recombinant proteins of E2F1 were incubated with either S^35^-labeled (top) or ectopically expressed myc-EBNA3C in HEK293 lysate (bottom). After preclearing of all S^35^-radiolabeled translated proteins with GST-beads for 1 h at 4°C, samples were subjected to GST-pull-down by incubating with either recombinant GST alone or wild-type GST-E2F1 protein as indicated. Samples were electrophoretically separated on 8% SDS-PAGE and were subjected to either autoradiography or western blot using anti-myc antibody. A parallel coomassie stained 12% SDS-PAGE resolving purified GST-proteins is shown at the bottom panel of B. C) The schematic illustrates different structural and interaction domains of E2F1 and summarizes the binding studies among EBNA3C and E2F1. +, binding; −, no binding. NLS, nuclear localization signal; DBD, DNA binding domain; LZ, leucine zipper motif; MB, Marked box. The asterisk indicates the immunoglobulin bands.

E2F1 consists of many domains, including the N-terminal Cyclin A binding domain, DNA binding domain (DBD), dimerization domain, and a C-terminal transactivation domain [2], [62]. The pRb binding region is located at the transactivation domain within residues 400–437 [2]. To identify the domains of E2F1 that are required for binding to EBNA3C, an *in vivo* binding assay was performed by immunoprecipitating flag-tagged E2F1 expression constructs encoding various domains of E2F1 with myc-tagged EBNA3C protein. The co-immunoprecipitation of EBNA3C was detected using anti-myc antibody. The data presented in Figure 3A showed that the E2F1 residues 1–437, 1–400, 1–310 and 1–243 strongly bound to EBNA3C, whereas the C-terminal region of E2F1 residues 243–437 did not bind to EBNA3C. In order to corroborate this binding experiment a subsequent GST-pull down experiment was carried out by incubating in vitro translated radio-labeled EBNA3C with different GST-fused E2F1 proteins, including residues 1–437, 1–310, 1–243, 1–150 and 243–437 (Figure 3B). The results indicated that the EBNA3C binding region within E2F1 lies predominantly within the N-terminal amino acids 1–243 of E2F1 containing Cyclin A and DNA binding domains (Figure 3C).

### EBNA3C inhibits E2F1 transcriptional activity

**Figure 4:**
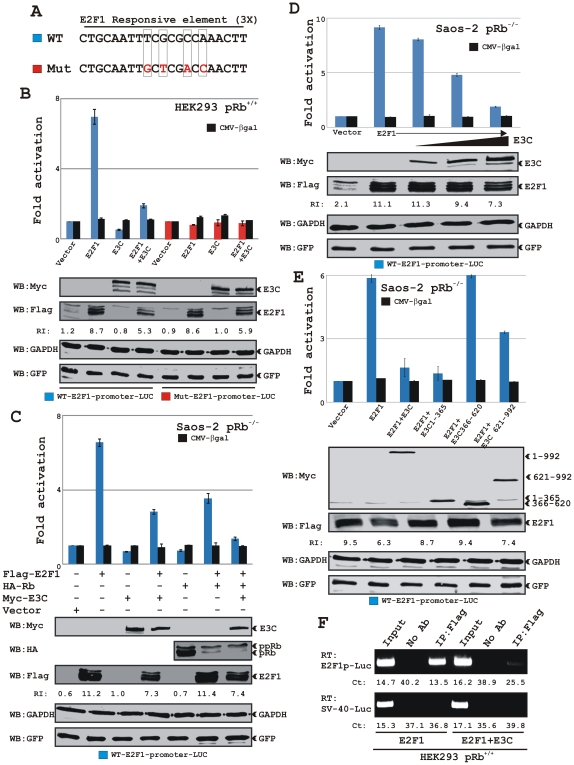
Co-expression of EBNA3C blocks E2F1 mediated transcriptional activity. A) The schematic represents three wild-type (WT) and three mutant (Mut) copies of the E2F1 responsive promoter element fused with the luciferase gene in pGL2Basic. B) HEK 293 (pRb^+/+^) cells were co-transfected with either 10 µg of WT (blue) or Mut (red) E2F1 reporter plasmids in combinations of expression plasmids for myc-EBNA3C and flag-E2F1 as indicated. C–E) Saos-2 (pRb^−/−^) cells were transfected with WT E2F1 reporter plasmid in the presence of different expression constructs as indicated. Cells were additionally transfected with 5 µg of pCMV-βgal and pEGFP-C1 expression vectors to normalize transfection efficiency. At 36 h post-transfection, cells were harvested and lysed in reporter lysis buffer. Total amount of proteins were normalized by Bradford assay and both luciferase and β-galactosidase activities were measured as described in ‘Materials and Methods’ section. Mean values and standard deviations of three independent experiments are presented. Bottoms panels indicate a representative blot of 5% of the total cell lysates resolved by appropriate % SDS-PAGE demonstrating the expression levels of ectopically expressed proteins. GAPDH blot was done as an internal loading control. E2F1 protein bands were quantified using Odyssey imager software as indicated as arbitrary numerical values (relative intensity, RI) at the bottom of gel (B–E) based on both GFP and GAPDH loading controls. F) HEK293 cells transfected with the WT E2F1 reporter plasmid in the presence of either flag-E2F1 alone or flag-E2F1 plus myc-EBNA3C expressing plasmids were subjected to ChIP assay as described in ‘Materials and Methods’ section. The eluted DNA samples were subjected to qPCR analysis using primers directed for either E2F1-responsive promoter fused with luciferase gene (top) or SV-40 promoter region (bottom). Panels show representative pictures from two independent experiments.

The binding studies led us to investigate whether EBNA3C was capable of modulating E2F1 mediated transcriptional activity. To specifically test the transcriptional activity of E2F1 we used two different reporter plasmids containing three copies of either wild-type (3X-WT-E2F1-luc) or mutant (3X-Mut-E2F1-luc) E2F1 responsive sequence element cloned upstream of the luciferase gene (Figure 4A). HEK 293 (pRb^+/+^) cells were subsequently transfected with these reporter plasmids in the presence of either vector control or flag-tagged E2F1 with or without myc-tagged EBNA3C (Figure 4B). Cells were additionally trasfected with both GFP expression and β-galactosidase reporter constructs under CMV promoter in order to check transfection efficiency. The results clearly demonstrated that ectopic expression of E2F1 leads to transcription activation from the wild-type promoter, while the mutant promoter had no response (Figure 4B). Interestingly, when co-expressed with EBNA3C, the transcriptional activity of E2F1 was significantly reduced to more than 70% (Figure 4B). EBNA3C expression alone also showed a reduction in luciferace activity perhaps preventing endogenous E2F1 activity from accessing the wild-type promoter region, whereas it showed no activity on the mutant promoter, indicating the specificity of EBNA3C mediated E2F1 transcriptional repression (Figure 4B). β-Galactosidase activity was measured to evaluate equal transfection efficiency (Figure 4B). The expression levels of transiently expressed EBNA3C and E2F1 along with GAPDH as an internal loading control and GFP expression as a transfection efficiency control were analyzed by western blots (Figure 4B, bottom panels). The results showed that co-expression of EBNA3C led to a reproducible reduction in E2F1 expression level (Figure 4B), indicating that EBNA3C mediated repression of E2F1 transcriptional activity may also mediate through targeting E2F1 degradation.

Previous studies have indicated that pRb blocks E2F1 transcriptional activity by forming a stable complex with E2F1 [63]. In order to distinguish our results from the pRb effect, we conducted a similar experiment in a pRb null background using Saos-2 cells (pRb^−/−^) (Figure 4C). The reduction of E2F1 transactivation activity by EBNA3C was not pRb dependent, as the normalized luciferase activity presented in Figure 4C showed that in the presence of EBNA3C there was a reduction of E2F1 transcriptional activity by greater than 50% when compared to that of E2F1 alone (Figure 4C). Interestingly, EBNA3C co-operated with pRb to inhibit E2F1 transcriptional activity (Figure 4C). Moreover, increasing amounts of EBNA3C resulted in a dose-dependent inhibition of E2F1 transactivation in Saos-2 (pRb^−/−^) cell line (Figure 4D). Similarly, increasing amount of EBNA3C expression caused a gradual decrease in E2F1 expression levels without affecting GFP expression levels (Figure 4D, bottom panels). This further indicates that EBNA3C may regulate E2F1 stability besides affecting its transactivition process.

To further define the domain(s) of EBNA3C important for this activity and also to determine if the binding domain(s) of EBNA3C is essential for inhibition of E2F1-mediated transactivation, the reporter assays were extended using different truncated domains of EBNA3C (residues 1–365, 366–620 and 621–992). All truncated EBNA3C mutants were able to localize in nucleus since the wild-type EBNA3C contains three functional nuclear localization signals (NLS) located at residues 72–80, 412–418 and 939–945, respectively (Figure 2E) [64]. From the truncated EBNA3C mutants that were tested, only the N-terminal binding region of EBNA3C (residues 1–365) showed an almost similar ability to repress E2F1-dependent transcriptional activity as of wild-type EBNA3C (Figure 4E). The C-terminal binding domain (residues 621–992) showed an approximately 50% activity when compared to either the full-length (residues 1–992) or the N-terminal binding domain (residues 1–365) (Figure 4E). However, the non-binding middle region (residues 366–620) of EBNA3C had no effect on E2F1 transcriptional activity (Figure 4E). Results from corresponding western blots indicated that co-expression of both full-length (residues 1–992) and the C-terminal binding domain (residues 621–992) led to a reduction in E2F1 expression levels but not in the presence of either N-terminal binding domain (residues 1–365) or non-binding middle region (residues 366–620) of EBNA3C (Figure 4E, bottom panels). Similarly, ectopic expression of EBNA3C proteins showed no effect on GFP expression, demonstrating that EBNA3C may regulate E2F1 transcriptional activity by multiple mechanisms.

Overall, the data suggest that EBNA3C represses the E2F1 transactivation activity by forming a complex with E2F1 at its N-terminal DNA binding domain, perhaps by interfering with its ability to access the target promoters. To test this hypothesis we performed a ChIP assay where we used a similar reporter plasmid containing 3X E2F1 responsive element transfected with vectors expressing flag-E2F1 with or without myc-EBNA3C in HEK 293 cells (Figure 4F). The ethidium bromide stained agarose gel of end products as well as the Ct values from real time PCR results showed that flag-E2F1 strongly bound to the E2F1 responsive sites, but this interaction was drastically impaired in the presence of EBNA3C expression (Figure 4F, top). The specificity of this experiment was confirmed by amplifying a similar size PCR product from the SV40 promoter region of the reporter plasmid, which showed no binding with E2F1 (Figure 4F, bottom). These findings demonstrate that EBNA3C efficiently blocks the recruitment of E2F1 to its responsive sites by inhibiting its DNA binding activity.

### EBNA3C inhibits E2F1 mediated anti-proliferative activities

It has been well established that in response to DNA damage E2F1 induces apoptosis through both p53-dependent and independent mechanisms [4]. The p53-dependent pathway is mediated through the activation of p19^ARF^ expression which eventually blocks Mdm2 activity [4]. On the other hand, the p53-independent pathway is mediated through the activation of pro-apoptotic genes including p73 and Apaf-1 [4], [9]. To specifically determine the significance of EBNA3C in terms of its direct regulation of E2F1 function, we wanted to verify whether or not EBNA3C could affect E2F1 mediated apoptosis. To investigate the regulation of E2F1 mediated apoptotic activity independent of p53, we used a p53-deficient cell line (Saos-2), since it has been shown earlier that the over-expression of E2F1 can lead to apoptosis in Saos-2 cells in response to DNA damage [65].

**Figure 5:**
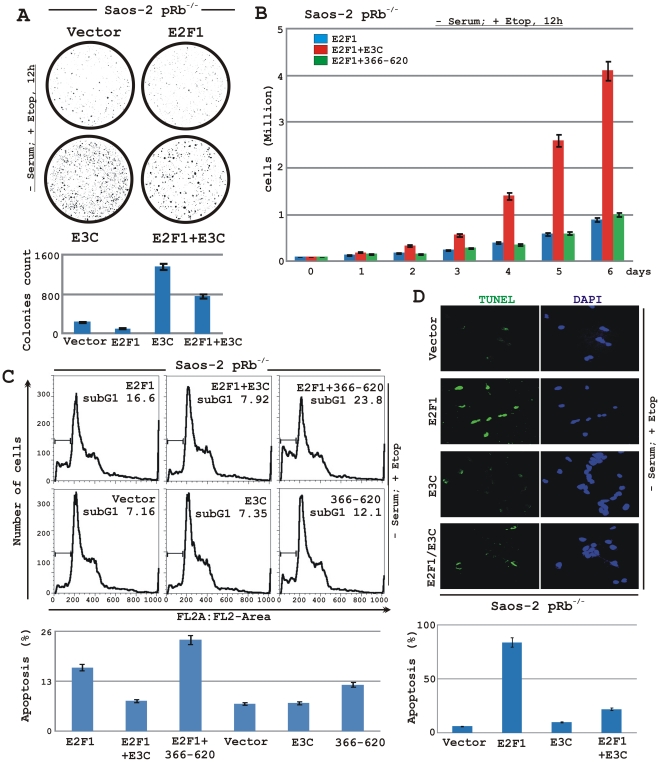
EBNA3C inhibits E2F1 mediated anti-proliferative activities in Saos-2 (p53^−/−^ pRb^−/−^) cells. A–D) Saos-2 (pRb^−/−^) cells were electroporated with expression plasmids for either empty vector, flag-E2F1, myc-EBNA3C or myc-EBNA3C residues 366–620 as indicated. A–C) Cells were additionally transfected with a GFP expression vector. At 24 h post-transfection cells were exposed to serum starvation and 5 µM etoposide treatment for 12 h, followed by selection with G418 for 2 weeks. A) After a 2-week selection, cells were fixed on the plates with 4% formaldehyde and scanned for GFP expressed colonies using Typhoon 9410 imaging system. The area of stained cells in each dish was calculated by Image J software. B) Approximately 0.1×10^6^ of flag-E2F1 and flag-E2F1 plus either full-length myc-EBNA3C or myc-EBNA3C residues 366–620 expressing selected cells from each set of samples were plated into each well of the 6-well plates and cultured for 6 days after 12 h treatment with serum starvation and 5 µM etoposide. Viable cells from each well were counted by trypan blue exclusion method daily using an automated cell counter. C) Selected cells with similar treatment as A) were subjected to flow cytometry analyses as described in ‘Materials and Methods’ section. Bar diagrams represent average sub G1 values of two independent experiments. D) Saos-2 cells transfected with different combinations of expression plasmids as described in panel A) and selected similarly as stated above with G418. After genotoxic stress with serum starvation and 5 µM etoposide treatment for 12 h cells were fixed and subjected for TUNEL assay as per manufactures protocol. A–D) All panels are representative of two independent experiments and bar diagrams represent the average data of two independent experiments with standard deviation.

First, to determine whether co-expression of EBNA3C can control the growth suppressive effects of E2F1 in response to DNA damage, we performed colony formation assays in Saos-2 cells (Figure 5A). Saos-2 cells were transfected with the expression plasmids for vector control, myc-tagged EBNA3C alone and flag-tagged E2F1 with or without myc-tagged EBNA3C (Figure 5A). Cells were additionally transfected with a GFP expression vector. After 24 h of transfection, cells were exposed to serum starvation plus etoposide (5 µM) treatment for 12 h, subsequently selected with G418 cultured in regular medium, and the number of colonies per plate was screened 14 days later. Figure 5A shows representative plates and average colony counts (bar diagram) from three independent experiments. The results showed that when Saos-2 cells were transfected with E2F1 alone; an approximately 2-fold reduction in efficiency of colony formation was observed compared to cells transfected with the empty vector (Figure 5A). However, cells co-transfected with E2F1 plus EBNA3C showed an approximately 7–8 fold increase in efficiency of colony formation compared to E2F1 alone (Figure 5A), indicating that EBNA3C expression neutralizes the growth inhibitory effect of E2F1 in response to initial DNA damage signals. Interestingly, EBNA3C alone exhibited a drastic effect in colony-formation efficiency compared to cells either expressing empty vector or E2F1 (Figure 5A).

In order to corroborate the previous experiment, we next performed a cell proliferation assay, where Saos-2 cells were transfected with plasmids expressing either flag-tagged E2F1 alone or in the presence of either myc-EBNA3C (wild-type, residues 1–992) or the non-binding EBNA3C domain (residues 366–620). After selection of the transfected cells with G418 similarly as stated above for 2 weeks, the proliferation rate of the selected cells was measured by an automated cell counter for 6 days (Figure 5B). Dead cells (approximately 5%) were excluded using Trypan Blue staining. The results showed that the cell-proliferation rate of cells stably expressing E2F1 plus EBNA3C was approximately 4-fold higher compared to either E2F1 alone or when co-expressed with EBNA3C residues 366–620 (Figure 5B). The results indicated that the interaction between EBNA3C and E2F1 is necessary to block E2F1-mediated anti-proliferative effects in response to DNA damage.

Can EBNA3C expression affect the ability of E2F1 to induce apoptosis? To answer this question, the levels of apoptotic cells in the stably transfected Saos-2 cells (as mentioned above) were examined in response to DNA damage signals by two successive methods, flow cytometric analysis and TUNEL assay (Figure 5C and 5D, respectively). Saos-2 cells stably expressing E2F1 resulted in induction of a significant level of apoptosis compared to the basal level of apoptosis in cells stably expressing either empty vector or EBNA3C alone (Figure 5C–D). However, co-expression of EBNA3C resulted in inhibition of E2F1 mediated apoptosis by approximately 50% (Figure 5C). This effect was more dramatic in TUNEL assays, with a decrease in approximately 75% (Figure 5D). However, in the presence of the non-binding region of EBNA3C (residues 366–620), there was no sign of reduction in E2F1-mediated apoptosis (Figure 5C). Instead there was a slight increase in the level of apoptosis (approximately 10%) (Figure 5C). Interestingly, the basal level of apoptosis of cells expressing EBNA3C residues 366–620 was relatively higher (approximately 5%) compared to both cells stably expressing vector control and wild-type EBNA3C (Figure 5C). Nevertheless, these data clearly indicated that EBNA3C can provide cells with a significant level of protection from E2F1 mediated apoptosis. Altogether our results suggest that EBNA3C plays a critical role in regulating the apoptotic and anti-proliferative functions of E2F1 independent of p53 in response to DNA damage.

### A reduction in EBNA3C levels enhances apoptotic cell-death in LCLs

**Figure 6:**
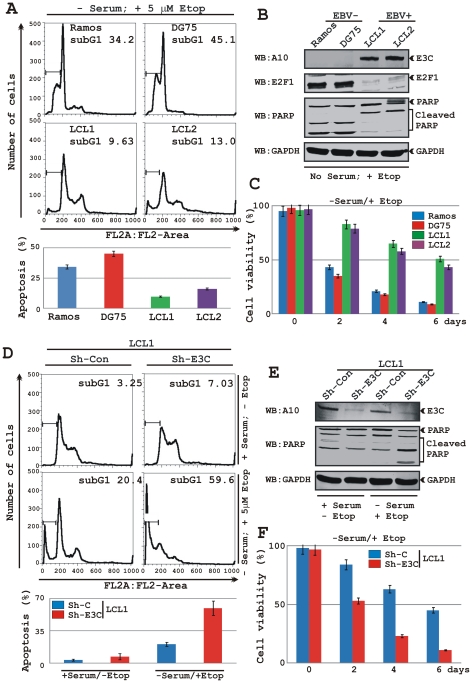
EBNA3C knockdown in LCLs leads to an increase in apoptotic cell death. A–C) Two EBV negative Burkitt's lymphoma lines - Ramos and DG75 and two EBV transformed cell lines - LCL1 and LCL2; D–F) Short hairpin (Sh) RNA mediated knockdown EBV transformed LCLs (Sh-Con or Sh-E3C) were subjected for genotoxic stress with serum starvation and 5 µM etoposide treatment for 12 h. A and D) Propidium iodide stained cells were analyzed by flow cytometry for a quantitative measurement of apoptosis (subG1 value). A and D) Bar diagrams below histograms represent the mean value of two independent experiments with standard deviation. B and E) Panels indicate representative western blots (WB) of 10% of the total cell lysates with indicated antibodies. C and F) Approximately 0.1 million of indicated cells were grown in 6 well plate for 6 days in RPMI medium containing 0.1% FBS plus 5 µM etoposide (−serum/+Etop) at 37°C. Viable cells from each well were counted by trypan blue exclusion method every 2nd day using an automated cell counter.

It has been shown earlier that EBNA3C blocks p53 dependent apoptosis [40], [42]. In addition, the abovementioned data clearly revealed that EBNA3C also negatively regulates E2F1 mediated apoptosis in a p53 null cell background. To determine the apoptotic cells in response to DNA damage signals, cells were cultured in medium with reduced serum (0.1% FBS) conditions and treated with 5 µM etoposide for 12 h prior to analyze by flow cytometry for sub G1 content (Figure 6A and 6D). Analysis of both serum starved and etoposide treated EBV negative Burkitt's lymphoma cells Ramos and DG75 showed an increased level of apoptotic cells compared to two different clones of LCLs (LCL1 and LCL2), which is approximately 3–4 fold higher (Figure 6A). To further test whether or not EBNA3C regulates the endogenous E2F1 activity in EBV transformed cells, LCLs knockdown for EBNA3C (Sh-E3C) were generated using lentiviruses that express short hairpin RNA against EBNA3C gene. LCLs with sh-control (Sh-Con) represent a non-complementary RNA element to the human genome sequence. As expected, reduction of EBNA3C expression in LCLs resulted in a significant increase in apoptosis (∼3-fold) compared to LCLs with sh-control in response to serum starvation and etoposide treatment (Figure 6D). In agreement to the flow cytometry results, western blot data also showed an elevated level of PARP cleavage in both EBV negative cell lines compared to EBV transformed LCLs (Figure 6B). As expected, EBNA3C knockdown LCLs revealed more PARP cleavage compared to LCLs with sh-control (Figure 6E). To further validate these observations, cell-death assays were conducted using these cell lines in the absence of growth stimuli for a period of 6 days (Figure 6C and 6F). The results showed that DNA damage caused by etoposide treatment and serum starvation resulted in a drastic increase in cell death (approximately a 4-fold difference) in EBNA3C knockdown cells (Sh-E3C) compared to wild-type (LCL1 and LCL2), as well as control LCLs (Sh-Con) (Figure 6C and 6F). These results further extends previously published data [66], [67], [68], which indicated that EBNA3C is absolutely necessary to block DNA damage response in LCLs.

### EBNA3C downmodulates p73 and Apaf-1 expression by inhibiting the DNA-binding ability of E2F1 to its targeted promoters in EBV transformed cells

**Figure 7:**
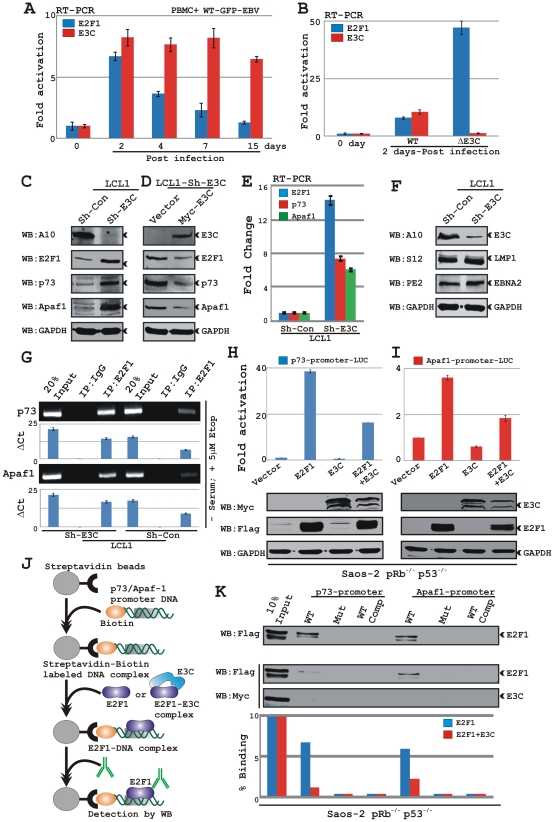
EBNA3C blocks p73 and Apaf-1 expressions by inhibiting the DNA-binding ability of E2F1 to its targeted promoters in LCLs. A–B) Approximately10 million human peripheral blood mononuclear cells (PBMC) were infected by either A) wild-type (WT) BAC GFP-EBV or EBNA3C knockout BAC GFP-EBV (ΔE3C) for 4 h. At indicates times post-infection cells were harvested, total RNA was isolated and subjected to cDNA preparation as per manufacturer's instruction followed by quantitative real-time PCR analysis for detecting *E2F1* and *EBNA3C* transcript levels. Each sample was tested in triplicate and data obtained from two independent experiments with two different donors and expressed as the difference of the quantity of specific transcripts to the quantity of GAPDH transcript as control. The fold change in expression of each mRNA relative to GAPDH transcript was calculated based on the threshold cycle (Ct) as 2^− Δ(ΔCt)^, where ΔCt = Ct_target_−Ct_GAPDH_ and Δ(ΔCt) = ΔCt_test sample_−ΔCt_control sample_. C) Approximately 10 million of EBNA3C and control knockdown LCLs were harvested and total cell proteins were subjected to western blot (WB) analysis using indicated antibodies. D) Approximately 20 million of EBNA3C knockdown LCLs were transfected with 50 ìg of plasmids expressing either vector control or myc-tagged EBNA3C via electroporation. Transfected LCLs were cultured in RPMI medium with 10% FBS for 48 h and subjected for western blot analysis using indicated antibodies. E) Total RNA was isolated from indicated LCLs, subjected to cDNA preparation as per manufacturer's instruction followed by quantitative real-time PCR analysis for detecting *E2F1, p73 and Apaf-1* transcript levels as similar to A–B). F) Approximately 10 million of EBNA3C and control knockdown LCLs were harvested and total cell proteins (50 µg) were subjected to western blot analysis using indicated antibodies. G) A ChIP assay was performed using either control or EBNA3C knockdown LCLs. Material immunoprecipitated with anti-E2F1 or control antibody (rabbit IgG) was amplified by using primers specific for p73 (top) or Apaf1 (bottom) promoters. The end products of qPCR bands ran into a 2.5% agarose gel. Bar diagrams represent the change in Ct value (ΔCt) over IgG. H–I) Saos-2 (p53^−/−^ pRb^−/−^) cells were transfected with either 5 µg of the wild-type H) p73-luciferase or I) Apaf-1-luciferase reporter plasmids with flag-E2F1 and myc-EBNA3C expression vectors as indicated. Luciferase activity was assessed at 36 h of post-transfection. J) Schematic representation of streptavidin pulldown assay as described in ‘Materials and Methods’ section. K) 100 µg of cell extracts from Saos-2 cells transfected with flag-E2F1 with or without myc-EBNA3C expression vector were incubated with 200 ng of the indicated biotinylated oligonucleotides (WT or Mut) immobilized with streptavidin accordingly to the manufacturer protocol, in the absence or presence of a 200 molar excess of the corresponding non biotinylated oligonucleotide (competition: comp). Oligonucleotide-bound E2F1 protein was detected by western blotting using anti-flag antibody (M2). The binding capacity of each oligonucleotide is given as percentage at bottom. All panels are representative of two independent experiments.

The abovementioned apoptotic phenomena due to downregulation of EBNA3C expression in LCLs could be attributed as a cumulative effect of both E2F1 and p53 mediated apoptosis. The inhibitory effect of EBNA3C on E2F1 mediated transcriptional activity led us to further investigate the basal expression levels of E2F1 both in primary as well as in latent infection model systems. For primary infection, approximately 10 million peripheral blood mononuclear cells (PBMC) from healthy donors were infected with either wild-type (WT) or EBNA3C knockout (ΔE3C) BAC-GFP EBV as previously described [22], [43]. In order to check EBV infection, GFP fluorescence was assessed using fluorescence microscopy (data not shown). Infected PBMCs with the wild-type virus were initially assessed for mRNA expression levels of both EBNA3C and E2F1 at different times of post-infection (0, 2, 4, 7 and 15 days) (Figure 7A). Real time PCR analysis demonstrated that EBNA3C activation typically occurred at 2 days post-infection and its expression was maintained at a constant level throughout the experiment, which was up to 15 days post-infection (Figure 1A). However, the E2F1 transcript levels was seen particularly robust at 2 days post-infection and gradually declined to a lower expression level similar to uninfected PBMC (Figure 1A). These data strongly corroborated the previous findings that the EBV-induced DNA damage response caused by an early period of hyperproliferation [67] is also linked to the cellular E2F1 expression level, which is further attenuated during LCL outgrowth. In order to determine a definitive role for EBNA3C in attenuating this E2F1 mediated DNA damage response, we generated EBNA3C knockout BAC-GFP virus and infected PBMCs for 2 days to analyze E2F1 transcript levels at hyperproliferative state (Figure 7B). Interestingly, EBNA3C knockout virus infected cells displayed a drastic increase (∼6–7 fold) in E2F1 activation compared to wild-type infection (Figure 7B). A similar trend, however to a lesser extent (∼2–3 fold) was observed 15 days post-infection (Figure S1C). These data clearly supported a concept that EBNA3C expression regulates genotoxic stress at the early stages of infection.

To further determine whether these changes also correlated in latent infection, we assessed both transcript and protein levels of E2F1 and its related apoptotic markers in EBNA3C knockdown LCLs. The results showed that knockdown of EBNA3C in LCLs resulted in an increased level of E2F1 expression both at the protein as well as transcript level, compared to control cells (Figure 7C and 7E, respectively), indicating that EBNA3C expression blocks E2F1 transcriptional activity in EBV transformed cells. Elevated expression of E2F1 also caused an enhanced level of E2F1 targeted gene expression, including p73 and Apaf-1 at the protein and mRNA levels (Figure 7C and 7E, respectively). Similarly, we also observed increased expression of Cyclin E, another bona-fide target of E2F1 during cell-cycle regulation, both at protein as well as transcript level (data not shown). This indicates the specificity of EBNA3C effect on E2F1 transcriptional activity, which is not only confined to E2F1 mediated apoptotic activities but also extended to E2F1 mediated cell-proliferation, possibly maintaining a feedback regulation of uncontrolled cell growth.

In order to corroborate this finding, the LCLs with EBNA3C knockdown cells were transiently transfected with plasmids expressing either vector control or myc-tagged EBNA3C. After 48 h of transfection, cells were harvested and subjected to western blot analysis (Figure 7B). The results showed that rescue of EBNA3C expression in EBNA3C knockdown LCLs had similar results to the wild-type (compare Figure 7C and 7D) as expression levels of E2F1, p73 and Apaf-1 were substantially reduced compared to cells with vector control (Figure 7D). Overall, these results clearly provide a possible explanation for the elevated level of apoptosis in EBNA3C knockdown LCLs. Since downregulation of EBNA3C in LCLs could affect the expression of other critical EBV latent proteins [69], we investigated the effect of EBNA3C inactivation on the expression of EBNA2 and LMP1 proteins using specific monoclonal antibodies. The results showed that the expression levels of both EBNA2 and LMP1 in EBNA3C knockdown LCLs were not affected when compared to control cells (Figure 7F).

To more rigorously evaluate the EBNA3C effect on E2F1 recruitment to its targeted promoters, we performed a ChIP assay on both endogenous p73 and Apaf-1 promoter using E2F1 antibody in these cell lines (both Sh-C and Sh-E3C). Indeed, the results showed that in response to DNA damage EBNA3C knockdown resulted in increased recruitment of E2F1 (2–3 fold) on these promoters compared to LCLs with Sh-control (Figure 7G). We next determined if the repressive effects of EBNA3C on the transcription level of both p73 and Apaf-1 seen in LCLs would also be observed using an exogenous system (Figure 7H–I). The results showed that in Saos-2 (pRb^−/−^) cells, co-transfection of both wild-type p73 and Apaf-1 promoters with an E2F1 expression vector resulted in activation of transcription, which was repressed by approximately 50% when co-expressed with EBNA3C (Figure 7H and 7I, respectively). Interestingly, EBNA3C expression alone also caused approximately 50% reduction of basal promoter activity (Figure 7H–I), perhaps by inhibiting the endogenous E2F1 activity in Saos-2 cells. It also suggested that EBNA3C may act more broadly to repress these promoter activities by blocking the recruitment of other transcription factors onto these promoters, as for example p53 recruitment on Apaf-1 promoter [70].

To address this phenomenon more directly and to nullify the effect from other transcriptional factors, we employed a direct oligo-pulldown assay where cell extracts from Saos-2 cells transfected with expression vectors for flag-tagged E2F1 with or without myc-tagged EBNA3C were incubated with biotinylated oligonucleotides containing only E2F1 responsive elements specific to either p73 or Apaf-1 promoters as described schematically in Figure 7J. Oligonucleotide-bound E2F1 protein was detected by immunoblotting using anti-flag antibody (Figure 7K). In agreement with the ChIP and reporter assays, these results also showed that the E2F1 DNA-binding activity to both oligonucleotides was hindered in the presence of EBNA3C (Figure 7K, compare the top and middle panels), providing a possible explanation for EBNA3C regulation of E2F1-mediated apoptosis. The specificity of this experiment was verified by using either mutant oligonucleotides or performing a competitive binding assay with 200 molar excess of the corresponding non-biotinylated oligonucleotide (Figure 7K). Taken altogether, these results showed that EBNA3C can block E2F1 mediated apoptosis by downregulating both p73 and Apaf-1 expression through inhibiting DNA-binding ability in EBV transformed cells.

### LCLs knockdown for E2F1 are less responsive to induction of apoptosis

**Figure 8:**
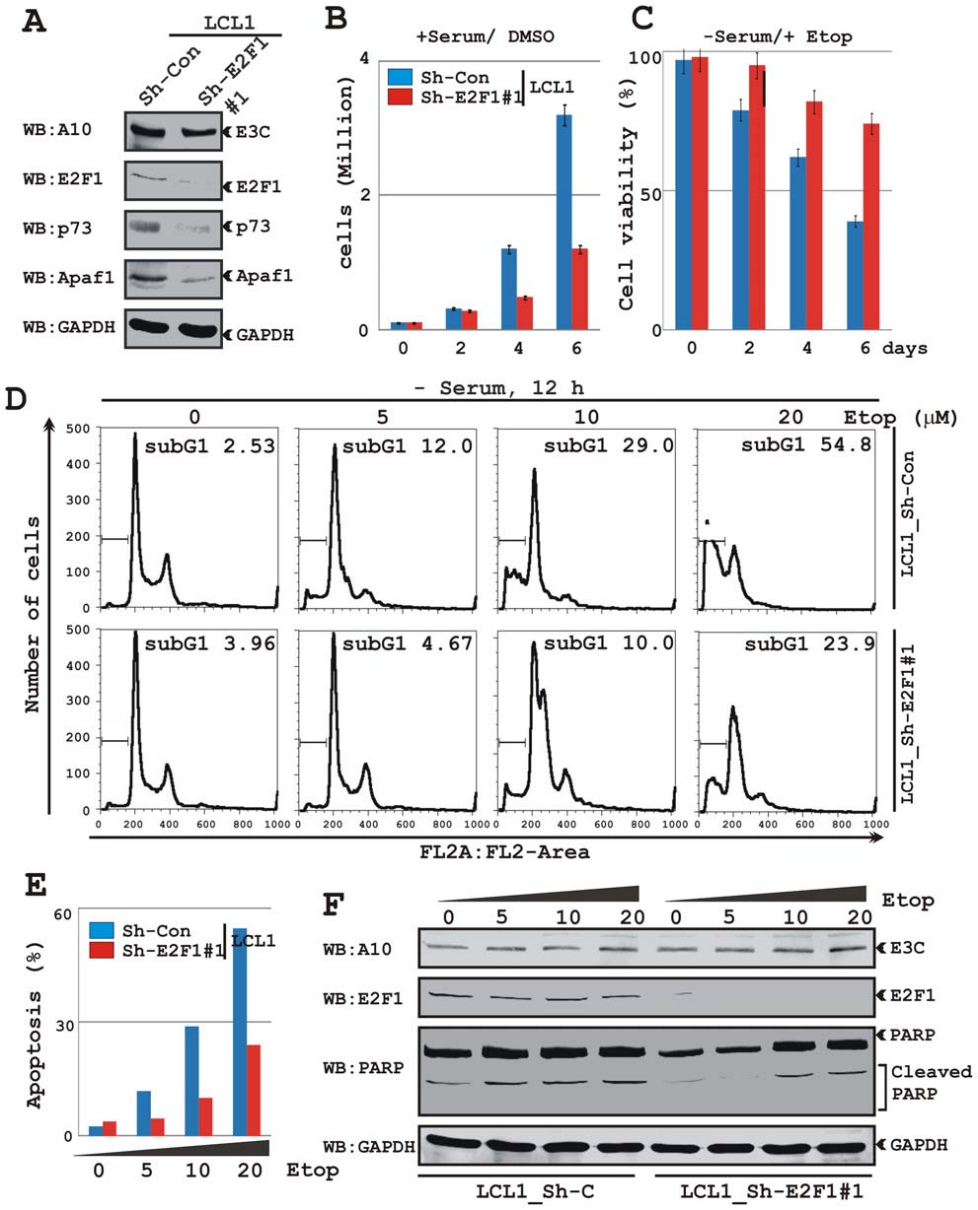
LCLs with E2F1 knockdown are less responsive to apoptosis. A) Approximately 10 million of Sh-RNA directed control or E2F1 knock-down LCLs (Sh-Con and Sh-E2F1 #1, respectively) were harvested and total proteins were subjected to western blot (WB) analysis using indicated antibodies. B–C) Approximately 0.1 million of indicated cells were grown in 6 well plate for 6 days in RPMI medium containing either B) 10% FBS (+serum/DMSO) or C) 0.1% FBS plus 5 µM etoposide (−serum/+Etop) at 37°C. Viable cells from each well were counted by trypan blue exclusion method every 2nd day using an automated cell counter. D) Cells were treated with serum starvation and an increasing dose of etoposide (0, 5, 10 and 20 µM) for 12 h, harvested, stained with propidium iodide and analyzed by flow cytometry. E) The bar diagram represents the change in G0 phase due to serum starvation and etoposide treatment in D). F) In a parallel experiment similar as D) approximately 2 million of indicated cells were subjected for western blot to detect the endogenous expression levels of EBNA3C, E2F1, PARP and GAPDH.

To further assess E2F1 mediated apoptotic activities in EBV transformed cells, we generated LCLs stably knockdown for E2F1 (Sh-E2F1) using similar lentivirus technique as mentioned before. In order to minimize the off-target effects we chose two different Sh-RNA sequences which are previously reported [47]. First, LCLs were transiently transfected with these Sh-RNA containing plasmids and validated by western blot 48 h post-transfection (Figure S1D). The results showed that both these Sh-RNAs efficiently silenced E2F1 expression in transfected LCLs (Figure S1D). Subsequently, corresponding lentiviruses were made from Sh-E2F1 #1 expressing vector and stably trasfected LCLs were generated. As expected, western blot analysis of these cells showed that reduction of E2F1 level also resulted in a significant decrease in expression levels of E2F1 regulated apoptotic markers including both p73 and Apaf-1 as well as cell-cycle regulatory protein Cyclin E (Figure 8A). This indicates that downregulation of E2F1 may have an effect on both cell-proliferation and apoptosis in EBV transformed cells.

To further explore E2F1 function in regulating LCLs growth, proliferation assays were conducted both in the absence and presence of DNA damage response (Figure 8B and 8C, respectively). Interestingly, the results showed that upon E2F1 knockdown, LCLs response to varying stimuli was distinctly different (Figure 8B–C). As expected from the western blot results (Figure 8A), the growth rate of LCLs knockdown for E2F1 (Sh-E2F1 #1) showed a significant reduction (approximately 2-fold) compared to control cells (Sh-Con) in the presence of mitogenic stimuli (normal cultured medium with growth factors) (Figure 8B). Importantly, the cell cultures used in these assays had greater than 98% viability, as determined by trypan blue exclusion method (data not shown). However, in the absence of growth stimuli, etoposide (5 µM) treatment induced marked cell death in control cells (Sh-Con) than that of E2F1 knockdown LCLs (Sh-E2F1 #1) over a period of 6 days, probably by inducing a greater level of apoptosis (Figure 8C). To further support this notion, we performed an apoptosis assay to quantitatively determine the apoptotic response in these cell lines with an increasing concentration of etoposide (Figure 8D–F). The representative histogram shows the analysis of multiple experiments which clearly demonstrated that LCLs knockdown for E2F1 (Sh-E2F1 #1) were less responsive (approximately 2-fold) to apoptotic stimuli compared to control cells (Sh-Con) as seen with an increasing dose of etoposide treatment (Figure 8D–E). Western blot of PARP cleavage in these cells additionally confirmed the flow-cytometric analysis, which showed more cleavage in control cells (Sh-Con) compared to E2F1 knockdown LCLs (Sh-E2F1 #1) in a dose dependent manner (Figure 8F). These observations clearly suggest that E2F1 plays a dual role in EBV positive cells and the active engagement of EBNA3C and E2F1 is necessary to block E2F1 induced apoptosis in response to DNA damage signals in LCLs.

### EBNA3C destabilizes E2F1 through an ubiquitin-proteasome dependent pathway

**Figure 9:**
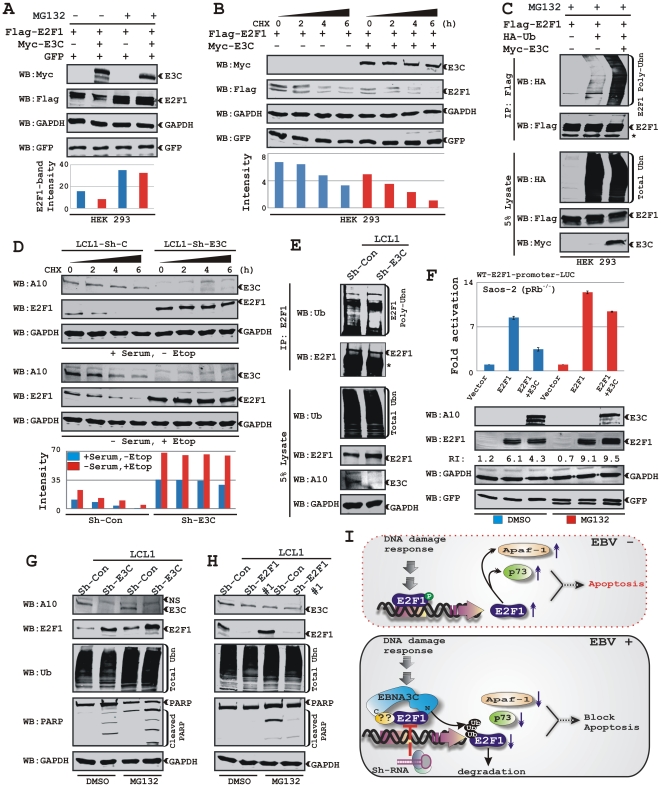
EBNA3C expression leads to E2F1 destabilization via an ubiquitin-proteasome dependent pathway. A) HEK 293 cells were co-transfected with flag-E2F1 and either vector control (lanes 1 and 3) or myc-EBNA3C (lanes 2 and 4) with GFP expressing vector. At 36 h posttransfection, samples were treated with either 20 µM MG132 (+lanes) or DMSO (−lanes) for 6 h and resolved by 10% SDS-PAGE and probed with the indicated antibodies. B) HEK 293 cells were similarly transfected with expression plasmids for flag-E2F1, myc-tagged EBNA3C and GFP plasmids as indicated. At 36 h post-transfection, cells were treated with 40 µg/ml cyclohexamide (CHX) for indicated lengths of time. 5% of the lysate from each sample were resolved by 9% SDS-PAGE and western blotted with indicated antibodies. C) 15 million HEK 293 cells transfected with different combinations of expression plasmids as indicated. Cells were harvested at 36 h, and total protein was immunoprecipitated (IP) with flag antibody and samples were resolved by 9% SDS-PAGE. D) Approximately 10 million of stably generated LCLs with either Sh-control (Sh-Con) or Sh-EBNA3C (Sh-E3C) were incubated with 100 µg/ml CHX for indicated lengths of time in RPMI medium containing either 10% FBS (+serum/DMSO) or 0.1% FBS plus 5 µM etoposide (−serum/+Etop) at 37°C. 10% of the lysate from each sample were resolved by 9% SDS-PAGE and western blotted with indicated antibodies. E) Approximately 50 million LCLs with either control Sh-RNA (Sh-Con) or EBNA3C directed Sh-RNA (Sh-E3C) were harvested after 10 h incubation with proteasome inhibitor MG132 (40 µM). Cells were lysed and E2F1 was immunoprecipitated (IP). Samples were resolved by 9% SDS-PAGE and western blotting (WB) was done by stripping and reprobing the same membrane. F) Saos-2 cells transfected with the WT E2F1 reporter plasmid in the presence of either flag-E2F1 alone or flag-E2F1 plus myc-EBNA3C expressing plasmids followed by incubated with either DMSO or MG132 (20 µM) were subjected for reporter assay as essentially described in ‘Materials and Methods’. Mean values and standard deviations of three independent experiments are presented. Bottoms panels indicate a representative blot of 5% of the total cell lysates resolved by appropriate % SDS-PAGE demonstrating the expression levels of ectopically expressed proteins. E2F1 protein bands were quantified using Odyssey imager software as indicated as either bar diagrams (A, B and D) or arbitrary numerical values (relative intensity, RI) at the bottom of gel (B–E) based on GFP or GAPDH loading controls, where applicable. G–H) Approximately 10 million of indicated cells incubated with either DMSO or MG132 (40 µM) for 10 h, harvested and 10% of total lysates were subjected for western blots with indicated antibodies. For all western blots, where appropriate, GAPDH serves as an internal loading control and GFP as a transfection efficiency control. Western blotting was done by stripping and reprobing the same membrane. Protein bands were quantified using Odyssey imager software as indicated either as arbitrary numerical values at the bottom of gel or as bar diagrams based on either GAPDH or GFP loading control. I) In response to DNA damage signals E2F1 gets stabilized and transcriptionally activates pro-apoptotic genes p73 and Apaf-1, which eventually induces apoptosis. In EBV transformed cells, by forming a stable complex with E2F1, EBNA3C inhibits its DNA binding activity and inhibits the transcriptional activation of p73 and Apaf-1. Moreover, EBNA3C specifically targets E2F1 for an ubiquitin-proteasome mediated degradation, which altogether blocks apoptotic induction. Moreover, sh-RNA designed against E2F1 showed reverse consequences and augments the apoptotic resistance of the cells.

E2F1 expression is strictly cell-cycle dependent and its protein level is unstable due to active degradation through the ubiquitin–proteasome pathway [71]. So far, our results showed that EBNA3C expression led to a decrease in steady state level of E2F1 expression. One mechanism, which we clearly demonstrated was that EBNA3C can efficiently block E2F1 mediated transcription. Since, EBNA3C was earlier shown to play a critical role in modulating the ubiquitin-proteasome machinery to regulate many important cell-cycle components [41], [43], [54], [60], we wanted to further determine whether or not EBNA3C can also regulate E2F1 degradation. To examine our hypothesis, transiently transfected HEK 293 cells with flag-tagged E2F1 with or without EBNA3C were treated with the proteasome inhibitor, MG132 (Figure 9A). Cells were additionally transfected with GFP expression plasmid to check the transfection efficiency. The results showed that co-expression of EBNA3C led to a considerable decrease in E2F1 ectopic expression levels (∼1.5 fold), whereas no change was observed in GFP expression levels (Figure 9A). However, after treatment with MG132 for a period of 12 h, the loss of E2F1 expression level was rescued compared to mock treatment. This strongly indicates that the decreased level of E2F1 observed in the presence of EBNA3C was a result of destabilization of E2F1 through the ubiquitin-proteasome degradation pathway.

To directly assess EBNA3C mediated destabilization of E2F1, HEK 293 cells were transfected with flag-E2F1, myc-EBNA3C and GFP expression vectors. At 36 h post-transfection, cells were treated with protein synthesis inhibitor cycloheximide (CHX), and samples were collected at different intervals - 0, 2, 4, and 6 hours. Western blots probed with flag antibody showed that the stability of the E2F1 protein was significantly reduced by EBNA3C co-expression, whereas GFP expression was unaltered (Figure 9B). The decreased stability of E2F1 in the presence of EBNA3C, prompted us to investigate whether EBNA3C facilitates poly-ubiquitination of E2F1 and thus enhances its degradation. To explore this possibility, a ubiquitination experiment was set up, where HEK 293 cells were transiently co-transfected with expression constructs for HA-ubiquitin, flag-E2F1 and myc-EBNA3C and the ubiquitination of E2F1 was measured by immunoprecipitation followed by Western blotting with anti-HA antibody (Figure 9C). The results clearly demonstrated a significant elevation in E2F1 poly-ubiquitination level in the presence of both EBNA3C and ubiquitin (Figure 9C). Overall, the results of these experiments suggest that EBNA3C can destabilize E2F1 by regulating its targeted degradation likely through recruitment of the ubiquitin-proteasome degradation system.

To more rigorously assess this in an endogenous background, we analyzed E2F1 stability as well as its ubiquitination levels using EBNA3C knockdown LCLs (Figure 9D and 9E). The results showed that in wild-type LCLs (Sh-Con), E2F1 was degraded to near completion by 4 h, whereas in EBNA3C knockdown LCLs, E2F1 stability was significantly extended after addition of CHX (Figure 9D). E2F1 half-life was determined to be ∼2 h in EBNA3C expressing wild-type LCLs; however, it was noticeably extended to more than 6 h when EBNA3C expression was compromised (Figure 9C). The results also indicated that in the presence of DNA damage signals (reduced serum and etoposide treatment) caused a significant increase in E2F1 expression as well as its stability in both control as well as EBNA3C knockdown LCLs (Figure 9D). Interestingly, EBNA3C stability also seem to be affected in response to DNA damage signals compared to mitogenic stimuli, where no sign of EBNA3C degradation was observed (Figure 9D, compare panels 1 and 3). The consequences of the transient decrease in EBNA3C level in response to DNA damage was thus manifested in an increase of E2F1 stability, which may explain the E2F1 dependent apoptotic phenomenon in EBV transformed LCLs. As expected, western blot analysis with anti-ubiquitin antibody of immunoprecipitated E2F1 revealed a significant decrease in ladder of higher molecular weight E2F1 species in EBNA3C knockdown LCLs compared to control cells (Figure 9E). This ladder is even more evident with MG132 treatment (data not shown).

The dynamic changes in E2F1 expression level in the presence of EBNA3C and MG132 treatment support our reporter assays above indicating that ubiquitin-proteasome dependent degradation is also associated with EBNA3C mediated E2F1 transcriptional suppression that is attenuated in response to DNA damage signals. To determine whether these changes correlated with E2F1 transcriptional activity, we performed similar promoter assays as described before using wild-type (3X-WT-E2F1-luc) E2F1 responsive reporter construct in the absence and presence of MG132 (Figure 9F). The results showed that addition of MG132 caused an inhibition of EBNA3C mediated blocking of E2F1 transcriptional activity (Figure 9F), which was also evident from E2F1 western blots (Figure 9F, bottom panels). However, MG132 addition could not completely reverse EBNA3C mediated E2F1 transcriptional inhibition, suggesting that even if the total E2F1 protein was enhanced via deregulation of the ubiquitin-proteasome machinery, free E2F1 species was still scarce due to the active EBNA3C-E2F1 complex. We next assessed whether MG132-mediated activation of E2F1 transcriptional activity affects apoptotic regulation in LCLs (Figure 9G–H). The presence of MG132 led to an increase in E2F1 total protein levels in both control as well as EBNA3C knockdown LCLs causing a considerable elevation in apoptosis as evident from PRAP cleavage (Figure 9G). However, the extent of apoptosis was much lower in control cells compared to EBNA3C knockdown LCLs (Figure 9G). Importantly, MG132 treatment did not show any significant effect in LCLs silenced for E2F1 (Figure 9H), suggesting that MG132 specifically acts to alleviate E2F1 protein stability through blocking of the ubiquitin-proteasome degradation pathway rather than via a different mechanisms. Overall the data suggest that besides blocking E2F1 transcriptional activity, EBNA3C actively participates to regulate E2F1 degradation in a ubiquitin-proteasome dependent manner (Figure 9I).

## Discussion

Cancer development critically depends on the subtle balance between cell proliferation and apoptosis mediated cell death. p16^INK4a^-Cyclin D/CDK-Rb-E2F cascade is thought to be a major determinant in regulating cell fate. Deregulation of E2F family member activities occurs due to the functional deviation of the upstream molecules in this pathway, which includes inactivation of Rb pocket proteins (pRb, p107, p130), p16^INK4a^ tumor suppressive functions, genetic manipulation of *cyclin D* (*D1*, *D2* and *D3*) oncogenes and its kinase partners CDK4/6, which confers a growth advantage and thus has become a hallmark of human cancer [72], [73]. In addition to regulation of cell proliferation, compelling evidence now indicates that E2F1 can also induce apoptosis under various cellular events regardless of p53 status [9], [47], [53]. Given the frequent inactivation of the tumor suppressor proteins pRb and p53 in human cancers [74], [75], E2F1 mediated apoptosis may provide an additional tumor surveillance mechanism. The E2F1 mediated apoptosis pathway is therefore emerging as a promising therapeutic target in controlling cancer development [2], [76].

Previous reports have suggested that the EBV essential latent antigen EBNA3C critically manipulates upstream components of E2F1 in this pathway. For example, EBNA3C mediated repression of p16^INK4a^ expression was shown to be essential for LCLs growth [23], [77]. Recently, we have shown that EBNA3C facilitates S phase entry through stabilizing and enhancing Cyclin D1/CDK6 activity [43]. Moreover, EBNA3C recruits SCFSkp2 E3 ligase activity for ubiquitin-mediated degradation of pRb [54]. EBNA3C was also shown to interact with pRb in the presence of proteasome inhibitor [54]. It is therefore compelling to investigate whether EBNA3C can further regulate the function of E2F1; the downstream effector of this pathway in order to control proliferation of EBV associated cancer cells. We initiated our study with binding experiments and we conclusively show that EBNA3C and E2F1 can form a pRb independent complex. Using a series of truncated EBNA3C and E2F1 proteins, we show that the N-terminal DNA binding domain of E2F1 (residues 1–243) is sufficient to interact with two distinct sites of EBNA3C, one lies at N-terminal residues 100–200 and another at C-terminal region comprising residues 621–700. Earlier studies have shown that this N-terminal region of E2F1 is responsible for apoptotic induction but also contains a Cyclin A interaction motif [65], [78]. Interestingly, the N-terminal domain of EBNA3C binds to E2F1 directly, while the C-terminal domain associates in a complex with E2F1 in cells. This N-terminal binding region of EBNA3C was shown to be particularly important as it binds to many critical cell-cycle regulators, including Cyclin A, Cyclin D1 and pRb and SCF^Skp2^ [43], [54], [56], [60]. Furthermore, genetic study using recombinant EBV expressing conditionally active EBNA3C demonstrated the importance of this particular N-terminal domain of EBNA3C as upon deletion of this N-terminal region there was a significant reduction in LCLs growth [31], whereas the C-terminal domain (residues 621–700) was dispensable [31].

E2F1 is an essential transcriptional activator of many cellular genes required for the G1 to S phase transition [3], [7], [79]. The active participation of EBNA3C in controlling G1-S phase [43], [57], combined with our binding data, prompted us to investigate the feasibility of EBNA3C mediated regulation of E2F1 transcriptional activity. A recent study using a genetically engineered EBV Bacmid has also shown that EBNA3C strongly attenuates DNA damage response induced during EBV-mediated B-cell transformation [67]. In agreement with this data our results show that EBNA3C knockout virus is incapable of suppressing E2F1 mediated DNA damage response during the early stages of infection. Our results also show that EBNA3C represses E2F1 mediated transcriptional activity by blocking the E2F1-DNA binding ability in latent infection using EBNA3C knockdown LCLs as confirmed by endogenous ChIP experiments. Interestingly, in support of our finding a recent publication by White et al. also showed in a microarray analysis that E2F1 transcript is specifically elevated by EBNA3C knockout virus infection compared to wild-type EBV [80]. However, in this paper this observation is entirely ignored and unaccredited. In certain human tumors genetic amplification and over-expression of E2F3 has been documented, but there were no clear indication of an oncogenic role for the other ‘activators’ of the E2F family members (E2F1 and E2F2) [81]. In addition to its well-established function in controlling cell proliferation, E2F1 is also capable of DNA damage-induced apoptosis by targeting several related genes including p73, Apaf-1, and caspases [9], [12], [13], [14]. The data presented here allow us to propose a model in which association of EBNA3C with E2F1 inhibits its DNA-binding ability as well as transcriptional activity that eventually blocks E2F1 mediated apoptosis in response to DNA damage by downregulating the target genes p73 and Apaf-1.

A number of DNA damage signaling events are clearly involved in the induction of E2F1 and its stabilization. However, the mechanism by which these modifications can lead to E2F1 stabilization remains unclear. E2F1 protein is known to be regulated through an ubiquitin-proteasome pathway in a cell-cycle dependent manner [82], [83], [84], which relies upon its dissociation from pRb and its binding to specific E3-ubiquitin ligases. One of the E3-ubiquitin ligases involved in E2F1 ubiquitination and degradation is SCF^Skp2^ [85], [86]. As previously described EBNA3C was also shown to interact and recruit this E3 ligase activity for pRb degradation [54]. Thus, one can expect that EBNA3C may also be involved in regulating E2F1 protein stability through modulation of its ubiquitination status. Indeed, our results showed that EBNA3C facilitates E2F1 degradation in an ubiquitin-proteasome dependent manner. However, we are not certain whether EBNA3C recruits solely SCF^Skp2^ activity for E2F1 degradation, as there are a number of molecules which actively targets E2F1 for degradation [87]. Further a comprehensive study is required to evaluate the E2F1 degradation pathway in an EBV background. Our results support and extend our model in which two distinct events, the control of DNA binding and protein stability contribute to the downregulation of the transcriptional activation function of E2F1 in EBV transformed LCLs.

We could not rule out the contribution of other ‘unknown’ events that may control EBNA3C mediated inhibition of E2F1 induced apoptosis. For example, identification of ATM-Chk2 signaling pathway as a mediator that specifically stabilizes E2F1 through phosphorylation in response to DNA damage provides us a conceptual framework to understand the critical interplay between cell proliferation and apoptosis regulated by E2F1 [16], [17]. Specifically, Chk1 and Chk2 were shown to promote E2F1 stabilization and activity after genotoxic stress and thereby contribute to E2F1-induced upregulation of p73 and consequently apoptosis [52]. In addition, 14-3-3τ, a phosphoserine-binding protein, stabilizes E2F1 via inhibition of ubiquitination [15]. Interestingly, we have previously shown that EBNA3C targets Chk2 to bypass G2/M transition under genotoxic stress [68]. A recent study also showed that EBNA3C attenuated ATM-Chk2 DNA damage responsive signaling pathway to establish B-cell immortalization [67]. It would therefore be important to understand the precise molecular regulation of E2F1 induced apoptosis during initial as well as persistent EBV infection in primary B-lymphocytes. This is currently under investigation in our lab.

Beside protein phosphorylation, acetylation is also known to be a conserved mechanism modulating the activity of several pro-apoptotic proteins in response to DNA damage, so as to selectively induce apoptosis [9], [88]. It has been shown earlier that E2F1 post-translational modification that occurs after DNA damage is important in directing E2F1 on the promoter of the proapoptotic gene p73 [9], [88]. It is as yet unknown from our study whether EBNA3C can also affect E2F1 acetylation specifically in the presence of DNA damage signals to regulate apoptosis. Additional studies are required to fully elucidate the combinatorial effects of these different mechanisms and the intricate network by which EBNA3C affects both the levels and activity of E2F1 to regulate apoptosis.

It is well established that the ability of E2F1 to drive apoptosis is distinct from its ability to drive cell division [79]. Our data show a fascinating observation that E2F1 plays a dual role in EBV positive LCLs. As expected, LCLs knockdown for E2F1 exhibited a reduced growth rate, whereas, in response to DNA damage E2F1 knockdown LCLs were more resistant to apoptosis, indicating that E2F1 acts as both an oncogene and a tumor suppressor in response to different stimuli. It is possible that different E2F1 target genes were selectively modulated by the EBNA3C-E2F1 complex, during the cell-cycle and in response to DNA damage. However, this issue could not be directly addressed until the E2F1 target genes essential for both routes are clearly identified. The factors that determine the decisions of inducing either cell division or cell death need to be further investigated in EBV positive cells. Overall, our findings suggest that, in addition to its seemingly contradictory roles as oncogene and tumor suppressor in tumorigenesis, E2F1 actively promotes DNA-damage induced apoptosis in LCLs and thus it could serve as an important determinant for chemosensitivity in EBV associated human cancer therapy, irrespective of p53 status.

## Supporting Information

Figure S1 **EBNA3C deregulates E2F1 activity.** 50 million A) DG75 and two LCL clones (LCL1 and LCL2) and B) BJAB and two BJAB stable clones expressing EBNA3C (E3C7 and E3C10) were subjected to immunoprecipitation (IP) with EBNA3C specific mouse monoclonal antibody (A10). Samples were resolved by 9% SDS-PAGE and detected by western blot (WB) for the indicated proteins by stripping and reprobing the same membrane. C) Approximately 30 million human peripheral blood mononuclear cells (PBMC) were infected by either wild-type (WT) BAC GFP-EBV or EBNA3C knockout BAC GFP-EBV (ΔE3C) for 4 h. At 15 days post-infection cells total RNA was isolated from harvested cells, cDNA was prepared and subjected for quantitative real-time PCR analysis for detecting *E2F1* transcript level as described in Figure 7. D) Approximately 30 million of LCLs were transiently transfected with 50 µg of Sh-RNA expressing vectors as indicated by electroporation. Cells were harvested at 48 h post-transfection and subjected for western blot using indicated antibodies.
(TIF)
