# Supplementary figures and images for: E2F1 Mediated Apoptosis Induced by the DNA Damage Response Is Blocked by EBV Nuclear Antigen 3C in Lymphoblastoid Cells
Source: PLoS Pathog. 2012 Mar 15;8(3):e1002573. doi: 10.1371/journal.ppat.1002573 (PMC3305458; doi:10.1371/journal.ppat.1002573)

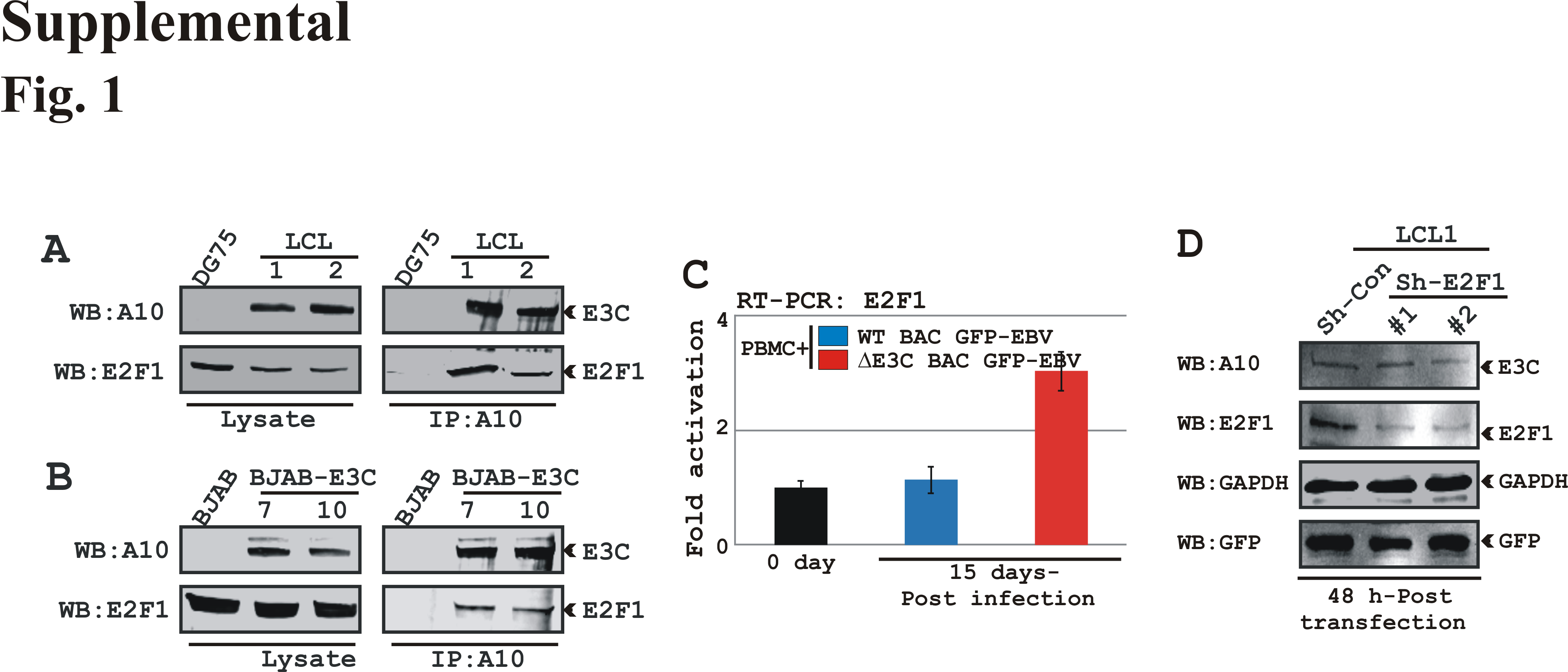

Supplement: Figure S1 — EBNA3C deregulates E2F1 activity. 50 million A) DG75 and two LCL clones (LCL1 and LCL2) and B) BJAB and two BJAB stable clones expressing EBNA3C (E3C7 and E3C10) were subjected to immunoprecipitation (IP) with EBNA3C specific mouse monoclonal antibody (A10). Samples were resolved by 9% SDS-PAGE and detected by western blot (WB) for the indicated proteins by stripping and reprobing the same membrane. C) Approximately 30 million human peripheral blood mononuclear cells (PBMC) were infected by either wild-type (WT) BAC GFP-EBV or EBNA3C knockout BAC GFP-EBV (ΔE3C) for 4 h. At 15 days post-infection cells total RNA was isolated from harvested cells, cDNA was prepared and subjected for quantitative real-time PCR analysis for detecting E2F1 transcript level as described in Figure 7. D) Approximately 30 million of LCLs were transiently transfected with 50 µg of Sh-RNA expressing vectors as indicated by electroporation. Cells were harvested at 48 h post-transfection and subjected for western blot using indicated antibodies. (TIF) [file ppat.1002573.s001.tif]
